# Origin of the clock in *Neurospora crassa*

**DOI:** 10.3389/fmolb.2025.1697003

**Published:** 2026-01-14

**Authors:** Ahmad Al-Omari, Cara Altimus, Jonathan Arnold, Sam Arsenault, Suchendra Bhandarkar, Shishir Bhusal, Christian Caranica, Jia Hwei Cheong, Zhaojie Deng, Arthur S. Edison, Garrett Floyd, James Griffith, Brooke Hull, Michael T. Judge, Yang Liu, Leidong Mao, Bijoy Mohanty, Xiao Qiu, H.-B. Schüttler, Ashley Scruse, Thiab Taha, Lingyun Wu, Yue Wu

**Affiliations:** 1 Biomedical Systems and Informatics Engineering Department, Yarmouk University, Irbid, Jordan; 2 Science Philanthropy Accelerator for Research and Collaboration (SPARC), Milken Institute, Washington, DC, United States; 3 Complex Carbohydrate Research Center, University of Georgia, Athens, GA, United States; 4 Department of Organismic and Evolutionary Biology, Harvard University, Cambridge, MASS, United States; 5 Department of Computer Science, University of Georgia, Athens, GA, United States; 6 Department of Physics and Astronomy, University of Georgia, Athens, GA, United States; 7 Center for Theoretical Biological Physics, Northeastern University, Boston, MASS, United States; 8 Rarecyte, Seattle, WA, United States; 9 Department of Molecular Biology, Princeton University, Princeton, NJ, United States; 10 Imperial College, London, United Kingdom; 11 College of Engineering, University of Georgia, Athens, GA, United States; 12 US Clinical Pharmacology and Pharmacometrics Department, Johnson and Johnson, Spring House, PA, United States; 13 Center for Broadening Participation in Computing, Morehouse College, Atlanta, GA, United States; 14 Department of Genetics, Stanford University, Stanford, CA, United States

**Keywords:** biological clock, coupled oscillators, microfluidics, continuous *in vivo* metabolism NMR, ensemble methods, stochastic resonance, stochastic coherence, quorum sensing

## Abstract

We examine the collective behavior of single cells in microbial systems to provide insights into the origin of the biological clock. Microfluidics has opened a window onto how single cells can synchronize their behavior. Four hypotheses are proposed to explain the origin of the clock from the synchronized behavior of single cells. These hypotheses depend on the presence or absence of a communication mechanism between the clocks in single cells and the presence or absence of a stochastic component in the clock mechanism. To test these models, we integrate physical models for the behavior of the clocks in single cells or filaments with new approaches to measuring clocks in single cells. As an example, we provide evidence for a quorum-sensing signal both with microfluidics experiments on single cells and with continuous *in vivo* metabolism NMR (CIVM-NMR). We also provide evidence for the stochastic component in clocks of single cells. Throughout this study, ensemble methods from statistical physics are used to characterize the clock at both the single-cell level and the macroscopic scale of 10^6^ cells.

## 1 Examples of collective behavior

Collective behavior can be observed in a variety of contexts (Sumpter, 2006; Sumpter, 2010; Gordon, 2023): the blinking of fireflies, the collective marching of an army of locusts (Buhl et al., 2006), the schooling of fish (Rosenthal et al., 2015), collective movement of baboons (Strandburg-Peshkin et al., 2015), the aggregation of social bacteria (Cotter et al., 2017), and the synchronized motion of birds in a flock (Ballerini et al., 2008). These kinds of collective behavior can also be observed at other levels of biological organization, such as the synchronized behavior of cells in tissues (Deng et al., 2016). Single cells, for example, have a biological clock (Deng et al., 2019), but their synchronized behavior is usually only observed at the level of 10^7^ cells (Larrondo et al., 2015). Even viral attacks are carried out as collectives (Erez et al., 2017). Collective behavior is a relatively new discipline (Gordon, 2023), but it has some substantial roots in insect social biology (Wilson, 1971).

Spectacular examples of collective behavior are schools of marine organisms, such as the Antarctic krill (Murphy et al., 2019). These schools can involve the synchronous movement of over 60 million individuals in a school. A fundamental question is how individuals in a school coordinate their behavior (Sumpter, 2010; Katz et al., 2011; Ashraf et al., 2017; Lavergne et al., 2019; Newbolt et al., 2019). A second challenge is tracking the whole school for the velocity and position of each member, particularly when there are over a million members in the school or flock (Ballerini et al., 2008; Cavagna et al., 2010). A major question is how the school protects its members and whether there are edge effects on avoiding predation (Jeschke and Tollrian, 2007; Duffield and Ioannou, 2017). Does this collective behavior also give them a collective intelligence to respond to environmental cues and avoid predation? Do they experience phase transitions from disorderly movement to highly organized schools and flocks (Vicsek et al., 1995)?

Another spectacular example of collective behavior are the marching armies of locusts that have plagued humankind since biblical times (Buhl et al., 2006). They affect crops on over 20% of the earth’s landmass. Once the marching army takes flight, it is very hard to control its damage to crops. Can one predict when a locust army aggregates and takes flight to begin its march (Buhl et al., 2006)? How do they march together? Some models for their coordinated marches have been developed (Buhl et al., 2006).

In addition to these two examples of collective behavior, two more examples of collective behavior at the cellular level are central to this review. The choice of organism is the filamentous fungus *Neurospora crassa* and its model circadian system. One example is the synchronized release of conidia by fungal filaments during filamentous growth along a glass tube filled with solid media (Baker et al., 2012). These are called “race tubes” as the filaments race down the tube, consuming fresh food in their path like marching locusts (Supplementary Figure S1A). The bands—made up of conidia—are spaced out and formed every 22 h as the filaments grow from one end to the other end of the race tube. This is the biological clock at work, controlling the asexual reproduction of the fungus, and the race tube is a standard screen for genes affecting circadian rhythms (Muñoz-Guzmán et al., 2021).

At an earlier life stage, this organism can also be grown in liquid culture containing only conidia with a fluorescent marker attached to a gene under clock control (Gooch et al., 2008). Two bursts of fluorescence can be seen over 48 h in the video (Supplementary Figure S1B). Most molecular research on the clock is done at this life stage in liquid culture studying only these two bursts (Larrondo et al., 2015). The clock can then be viewed as the collective behavior of cells synchronizing the phase of their individual cellular clocks either in fungal filaments or conidia. This study of single-cell behavior is a new focus of systems biology (Voit et al., 2023). All of these examples have one shared element: an underlying physical model to help explain how these remarkable populations transition from disorderly motion to synchronized dynamics (Vicsek et al., 1995). This is a continuous theme of this review. These models become tractable for study using ensemble methods from statistical physics (Landau et al., 2014).

## 2 Origin of the clock

The study of the clock began with the fruit fly and some help from the bread mold *Neurospora* (Hall, 2017). Research on the clock began with a yellow forked mutant isolated by Ed Lewis that led to his Nobel Prize on the genetics of development (Lewis, 1978). This yellow forked mutant was picked up by Seymour Benzer’s laboratory and used to carry out a systematic screen for genes controlling varied interesting behaviors (Benzer, 1973). One of these screens was performed by Ron Konopka as a graduate student in the Benzer laboratory that led to the capture of the first rhythmicity gene, *period* and others (Konopka and Benzer, 1971; Zehring et al., 1984). This research then gave rise to the interdisciplinary Nobel-Prize-winning study in 2017 by Jeffrey Hall, Michael Rosbash, and Michael Young (Hall, 2017). The characterization of these genes uncovered the mechanisms (Stoleru et al., 2004; Meyer et al., 2006) underlying the biological clock and its evolution in many organisms (Young and Kay, 2001), a phenomenon of general interest to all of us as we wake, eat, develop, reproduce, sleep, and age (Hall, 2017).

One of the exciting aspects of this research has been the mutant screens in two organisms: *Drosophila melanogaster* and *N. crassa*. The yellow fork screen led to the genes involved in the clock. In 1942, Beadle and Tatum, also at the same institution alongside the fly factory at Caltech, developed the first screen for biochemical mutations in genes, yielding the one-gene-one-enzyme hypothesis (Beadle and Tatum, 1941), for which they were awarded the Nobel Prize. This screen for biochemical function provided an approach that potentially allows an examination of how the clock originates at the single-cell level through the tools of metabolomics (Judge et al., 2019). The advantage of *N. crassa* in the study of circadian rhythms is that it has a mechanism known as “repeat-induced point” (RIP) mutation that ensures that most genes in *N. crassa* are single-copy (Selker, 1990), making the study of their function easier in *N. crassa* than in other systems.

In this simpler microbial context, it is possible now to examine how the clock behaves in single cells (Deng et al., 2016; Deng et al., 2019). It is natural to ask the fundamental collective behavior question: how do single cells (Deng et al., 2019) with their own clocks give rise to the biological clock as an emergent property that we all experience in our tissues and as an organism? In other words, how do the individual cells synchronize their time pieces (Sumpter, 2010)? This question on the collective behavior of single-cell oscillators is the focus of this review and fits into the larger common theme of collective behavior in explaining synchronized movement in terms of a phase transition.

**FIGURE 1:**
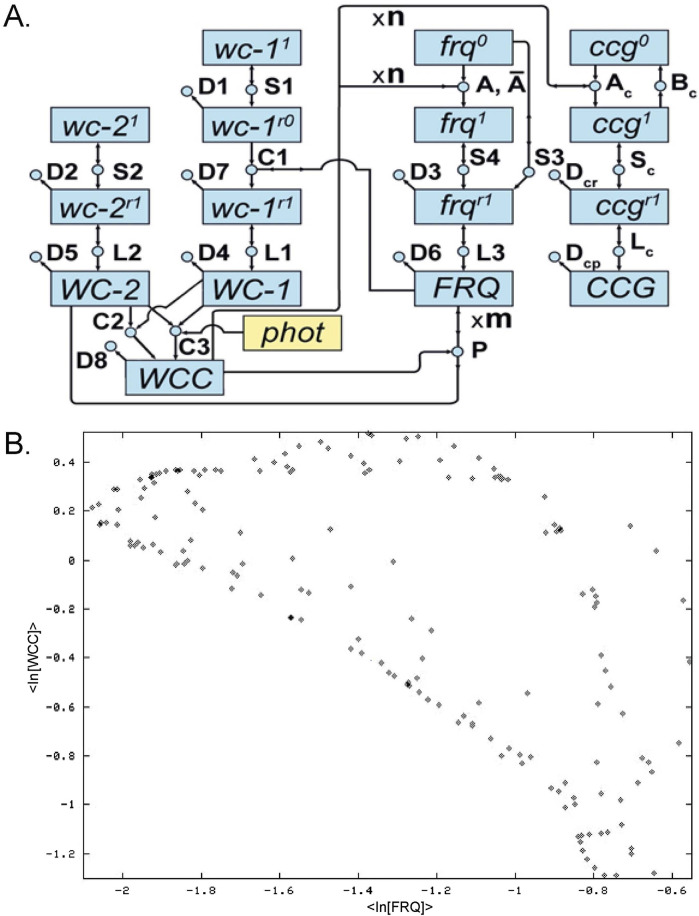
**(A)** Clock network on the macroscopic and microscopic scale. Circles are reactions. Boxes (labels inside) are molecular species. Rate constants, such as D6, are labels on reactions (Yu et al., 2007; Dong et al., 2008). n and m denote Hill coefficients. Capitalized box labels denote proteins. Box Labels with an r superscript are RNAs. Box labels with a 0 or 1 superscript denote genes as off or on. **(B)** Trace (Supplementary Video S3) of the ensemble fitted dynamics of the clock network in **(A)** computed to begin on 8/11/03 with n = m = 4. The expression of the oscillator FRQ protein (negative regulator) is shown on the x-axis, and the expression of WCC, the positive regulator of the oscillator, is shown on the y-axis.

As a foundation to addressing this question, one of the advantages of *N*. *crassa* as a kinetics model for time-keeping is that we have a good understanding of the mechanism (Cha et al., 2015) at both the macroscopic scale of millions or tens of millions of cells (Yu et al., 2007) and at the microscopic level of single cells (Deng et al., 2019; Caranica et al., 2020) (Figure 1A). We know the mechanism by which the organism tells time in a genetic network involving clock mechanism genes.

At the core of this network is the oscillator gene encoding the analog readout of the time of day—*frequency* (*frq*) (Aronson et al., 1994)—a homolog of the *period* gene (McClung et al., 1989). The *frq* gene is transcribed and translated into the protein FRQ, which reflects the time of day. At dusk, the FRQ protein is high in concentration, and at dawn it is low in concentration.

At the same time, two other genes in *N. crassa* represent regulators of the clock mechanism. The genes *white-collar-1* (*wc-1*) and *white-collar-2* (*wc-2*) encode proteins WC-1 and WC-2, which form the WCC = WC-1/WC-2 complex (Crosthwaite et al., 1997), which starts the oscillator, much like hand-pushing the pendulum on a grandfather clock. In turn, the resulting oscillator protein FRQ “bites the hand that feeds it” and deactivates the WCC, closing the negative feedback loop (Figure 1A). The FRQ protein appears to act as cyclin to recruit phosphatase/kinase pairs to degrade the WCC complex (Schafmeier et al., 2005). The FRQ oscillator constitutes the negative arm of the clock mechanism, while WC-1 and WC-2 constitute the positive arm of the clock mechanism (Dunlap, 1999). This negative feedback model in its deterministic form belongs to a family of models called “Hill-type transcriptional repression models” (Kim, 2016).

In contrast to *D. melanogaster* (Zoltowski et al., 2011), the light response of the *N. crassa* system is part of the positive activator, the WC-1 protein (Froehlich et al., 2002). The light response and transcription factor function is found in one gene product (WC-1) controlling both the expression of *frq* as well as serving as a blue-light receptor. Another important response of the clock is to temperature (Crosthwaite and Heintzen, 2010). While FRQ and VIVID appear to respond to temperature (Liu et al., 1997), there are no known receptors for temperature at this time (Crosthwaite and Heintzen, 2010).

Each of the macromolecules in the clock are connected by biochemical reactions, the circles in Figure 1A, which were the focus of Beadle and Tatum (1941) in their inspirational genetic study of metabolism. The squares are the macromolecules. The clock mechanism itself controls much of metabolism (Al-Omari et al., 2022) through the *clock-controlled genes* (Figure 1A). There are over 3,380 genes in the clock network, making over 30% of the *N. crassa* genome (Al-Omari et al., 2022). The dynamics of a fitted ensemble (Section 9) of models are shown in Figure 1B, with a 22 h intrinsic period when the system is running in the dark (D/D).

One of the themes of collective behavior is its mathematical foundation and grounding in physics (Sumpter, 2010). Understanding the behavior of coupled systems has been central to physics since its focus on orbits and coupled pendulums (Huygens, 1673; Oliveira and Melo, 2015) down to the present (Abbott et al., 2016). Sturtevant began his career with a mathematical problem of genetic mapping (Sturtevant, 1913) and encouraged (Sturtevant, 1929) Seymour Benzer (Hotta and Benzer, 1973) and others (Garcia-Bellido and Merriam, 1969) to examine the mathematical basis of the fate mapping of cell lineages’ controlling behavior to the *Drosophila* blastoderm (Merriam and Lange, 1974). Ultimately, it was Sturtevant’s academic offspring (Ed Lewis) who suggested a systematic search for mutants affecting key traits in development (Lewis, 1978) and eventually the clock under Seymour Benzer (Konopka and Benzer, 1971). In addition, both Young and Hall interacted with Benzer, a physicist by training, who were in turn receptive to mathematical approaches to behavior (Arnold and Kankel, 1981; Fox, 1986; Francois, 2005). The problem of understanding the synchronization of cellular oscillators in collective behavior (Sumpter, 2010) relies heavily on both physics (Caranica et al., 2020) and mathematics (O’Keeffe et al., 2017) for its understanding, as will become clear below.

## 3 Hypotheses of synchronization: How clocks synchronize?

There are four hypotheses considered here to explain the origin of the clock and the phase synchronization of single-cell oscillators giving rise to the biological clock in tissues and whole organisms (Table 1). The elements of these four hypotheses are the presence or absence of a stochastic component to the clock mechanism and the presence or absence of a communication mechanism between the clocks in single cells or filaments, known as “quorum sensing” (Miller and Bassler, 2001). First discovered in bacteria, quorum sensing is the regulation of gene expression in response to density (Miller and Bassler, 2001; Papenfort and Bassler, 2016; Whiteley et al., 2017). Well described quorum-sensing systems have been identified in bacteria, such as *Vibrio fischeri*, *Pseudomonas aeruginosa*, *Agrobacterium tumefaciens*, *Erwinia carotovora*, and cyanobacteria (Sharif et al., 2008). Evidence has also been presented for quorum sensing in the non-filamentous fungi *Candida albicans* (Chen et al., 2004; Hogan et al., 2004) and *Saccharomyces cerevisiae* (Chen and Fink, 2006). While quorum sensing has been reported in conidial anastomosis tubes (CATs) in the filamentous fungus *N. crassa*, no evidence was initially reported for the density-dependent response (Roca et al., 2005). Later research detailed the density-dependent response in CAT formation (Mehta and Baghela, 2021).

**TABLE 1:** Hypotheses about clock origin in a population of cellular oscillators.

	Hypotheses about clock origin	Models	
		No stochasticity	Stochasticity
Models	No quorum sensing	Neither QS nor SR: there is no quorum sensing or stochastic resonance available to explain the phase synchronization of cellular oscillators	Stochastic resonance (SR): there is an intermediate level of intrinsic cellular noise to explain the phase synchronization of cellular oscillators
	Quorum sensing (QS)	QS and no stochasticity: there is only QS to explain the phase synchronization of cellular oscillators	Stochastic coherence (SC): there is both QS and stochastic flipping on/off of the clock oscillator (*frq*) and quorum-sensing (*ccg*) genes to explain the phase synchronization of cellular oscillators

The other element of synchronization hypotheses is the role of stochasticity (Table 1). The importance of stochasticity in the formation of circadian rhythms was hypothesized by a specific mechanism called “stochastic resonance” (without data) (Sriram and Gopinathan, 2005) for *N. crassa*. Rappel and Strogatz (1994) illustrated a simple mechanism for how stochastic resonance could give rise to oscillations. In their model, there were two fixed points on a circle. Noise was introduced to push the system from one fixed point to the other. If there was not enough noise, the system would not leave the orbit of one fixed point and not produce oscillations. If there was too much noise, the system would wander freely between the two fixed points, not producing oscillations. If there was an intermediate level of stochastic noise (i.e., “stochastic resonance”), then oscillations would emerge. This is one of the stochastic mechanisms relevant for producing synchronization.

A reasonable null hypothesis is that neither stochasticity nor quorum sensing is present and available for the phase synchronization of cellular clocks (Table 1) because of the limited data on stochastic effects in *N. crassa* genetic networks and limited evidence for quorum sensing in filamentous fungi. Alternatively, quorum sensing could be present without stochasticity (Cheong et al., 2022) and lead to synchronization in phase of clocks in different cells, much as an atomic clock synchronizes our smart phones all over the world. In this analogy, the media containing different cellular oscillators is the equivalent of the atomic clock.

A second possibility is the presence of stochasticity in the system. Two hypotheses will be introduced that may explain the phase synchronization of cellular oscillators. The first is the aforementioned stochastic resonance hypothesis. This was first introduced in geophysics to examine global warming (Benzi et al., 1981) and is a physical hypothesis on how oscillations may arise in the presence of noise. Stochasticity may have other unexpected consequences, depending on the context (Table 1).

The second hypothesis, “stochastic coherence”, is related. Under this hypothesis there is stochastic switching on and off of key clock mechanism genes, such as the oscillator encoding gene *frq* and the *clock-controlled gene* (*ccg*), that control production of a quorum-sensing signal. Stochastic coherence differs from stochastic resonance in that, instead of pushing the system between two fixed points, it is switched between four deterministic mechanisms or models of circadian rhythms. The stochasticity is also limited to the transcriptional bursting in *frq* and the quorum-sensing gene *ccg*. This transcriptional bursting has been measured in a mammalian clock gene (Nicolas et al., 2018).

Each of these four hypotheses for phase synchronization, summarized in Table 1, will be examined in turn in the following sections of the review using a variety of screens for phase synchronization, including those both from genetics, biochemistry and microfluidics. Ensemble methods will be utilized in some cases to make predictions from a particular synchronization hypothesis.

## 4 Different cells march to a different drummer

**FIGURE 2:**
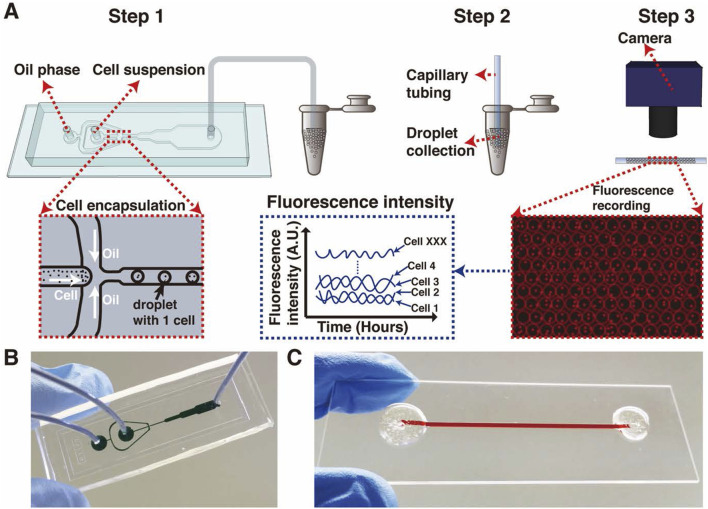
Droplet encapsulating microfluidic device from Deng et al. (2016) **(A)**. The dimensions of the two devices in **(B,C)** are that of a microscope slide, 75 mm x 25 mm.

To begin to understand the origin of the clock, it is necessary to know how cells tell time when they are isolated from each other. The use of droplet encapsulated microfluidics can be used to trap individual cells and observe their behavior in isolation (Deng et al., 2016) (Figure 2). The device is constructed with three channels. The middle channel contains cells and media; the side channels converge at the exit of the middle channel and contain oil. As cells move along the middle channel, the inflow of oil encapsulates them in droplets (Supplementary Figure S2). Thousands or tens of thousands of droplets are captured in a microfuge tube and transferred to a capillary tube in a single layer for viewing under a light microscope.

**FIGURE 3:**
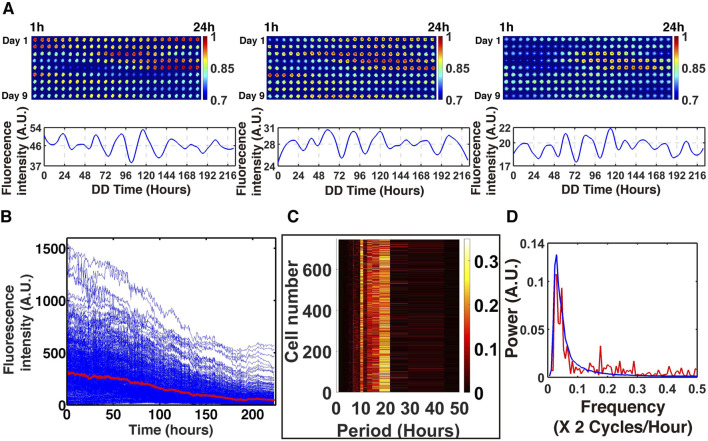
A total of 868 individual fluorescent trajectories in the droplet encapsulating device have circadian rhythms over 10 days (see randomly chosen trajectories in **(A)**), but the average of the fluorescent trajectories do not have circadian rhythms, probably due to phase cancellation **(B)** (Deng et al., 2016). A heat map of cell count versus the period of individual cells is shown with the most frequent period being between 20-22 h **(C)**. Also the average power spectrum of individual cells is reported with a strong peak at the frequency of .048 or period of 21 h **(D)**. DD denotes the cells were observed in the dark, and D/L, in the light. A. U. denotes arbitrary units.

The result is 868 isolated cells in droplets (Deng et al., 2019). The number of cells per droplet is stochastic, but the population of single isolated cells can be imaged as a subset of cells in droplets ranging from 1 to 16 (Figure 3). Figure 3A shows the randomly selected trajectories of three single cells. They clearly have circadian rhythms, but the rhythms have different phases. They do not have the same maxima and minima at the same times.

In panel 3b, all 868 isolated cell trajectories are plotted for their fluorescence over time. While individual cells may have oscillations, the average trajectory (in red) has no oscillatory signal. The phase differences of individual trajectories cancel each other out in the average trajectory. No signal emerges from isolated cells when their average behavior is examined. The trajectories of individual cells are noisy, and such noisy trajectories do strongly suggest the need for a physical model in which the basic measurements of counts of macromolecules in the cell are stochastic (Gillespie, 1977). These 868 “Gillespie” trajectories in Figure 3B were an impetus to develop a new ensemble method to fit stochastic networks (Caranica et al., 2020). Each of the 868 cells keeps time to a different drummer. To validate that they do have their own drumbeat, a power spectrum was constructed for each of these cells (Figure 3D). It can be seen that there is a strong peak at the frequency 0.048 or period of 21 h. The circadian rhythms can be measured over 10 days using the device. The use of microfluidics to observe circadian rhythms over 10 days in single cells is one of the fundamental screens used throughout our studies.

In contrast, if a liquid culture was observed to fluoresce or luminesce on a macroscopic scale of 10^7^ cells, then the circadian rhythms could only be seen for approximately 48 h (Supplementary Figure S1B), probably due to phase cancellation. The conclusion is that in the absence of communication, individual cells do have a circadian rhythm (Figures 3A,D), but it can only be observed by tracking the behavior of individual cells—in this case, in a microfluidics device to avoid phase cancellation across a population of cells.

In addition to establishing the circadian rhythms of these cellular oscillators, it was also necessary to confirm that these oscillators were light entrainable and held a stable period over a physiological range of temperatures—a property known as “temperature compensation” (Pittendrigh and Minis, 1972). These three properties of a true biological clock were established subsequently (Deng et al., 2019).

## 5 Evidence for synchronization

**FIGURE 4:**
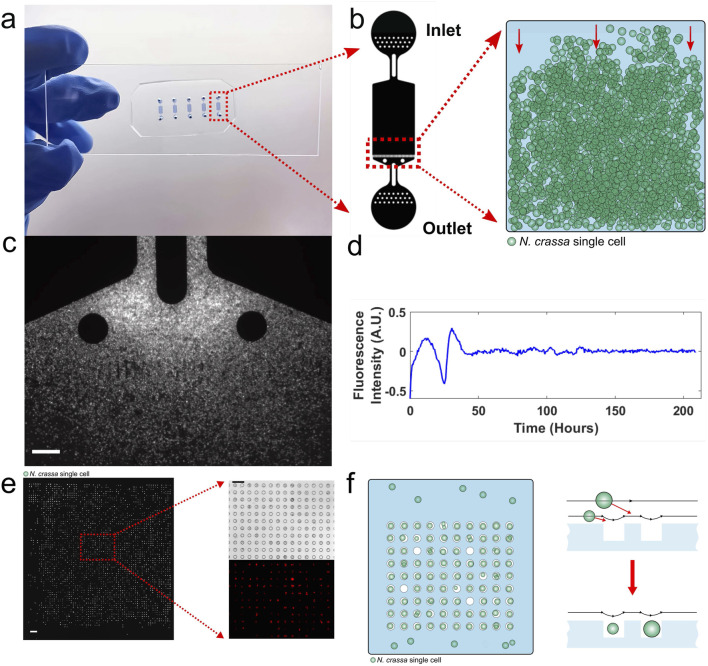
Big chamber and microwell devices are used to measure phase synchronization. **(a–c)** Big chamber device 1,800 × 1,150 × 10 microns (µm); scale bar is 50 µm. **(d)** Macroscopic limit achieved in the big chamber device, as in Supplementary Figure S1B. **(e,f)** Microwell device; scale bar is 100 µm on **(e)** (Cheong et al., 2022).

The hypothesis of coupled oscillators underlying circadian rhythms has a substantial history (Evans and Schwartz, 2024). For example, Pittendrigh (1960) hypothesized that there is both a morning and evening oscillator in eukaryotic systems (Evans and Schwartz, 2024). Some traction for this hypothesis is now available at the cellular level for a morning and evening oscillator in *Drosophila* (Stoleru et al., 2004). The question of coupled oscillators, however, cannot be avoided when beginning with cellular oscillators. We have shown that isolated cells that have no roommates displayed circadian rhythms (Deng et al., 2016), but they were not synchronized in these rhythms. What if the cells do have the opportunity to communicate? How does phase synchronization between the clocks in different cells change? To address this question, two new microfluidic devices were designed and built (Figure 4). One is called a “big chamber device” and the other, a “microwell device”.

The big chamber device acted to closely pack ∼150,000 conidial cells together in an artificial tissue. The question then is how they phase synchronize within this tissue (Figures 4a–c). The device is approximately 1,800 by 1,200 microns. Five fields of view were measured across a transect of the artificial tissue. The instantaneous phase (Caranica et al., 2019) was then compared between all pairs of fields of view (Supplementary Figure S3). In each comparison, the phase curves paralleled each other, indicating phase synchronization across the dimensions of the device.

One of the striking findings of this device was achieving the macroscopic limit to the fluorescent trajectories at ∼150,000 conidial cells in the big chamber device. The same average circadian behavior of two cycles at 10^7^ cells/mL in macroscopic liquid cultures was seen in the artificial tissue created by the big chamber device (Figure 4d). It is thus possible to duplicate macroscopic culture behavior with a microfluidics big chamber device.

Presuming that the hypothetical communication signal travels 1,800 microns by diffusion over 21 h, the radius of the communication molecule was estimated to be no more than 13 nm, which does not rule out a protein being a communication signal (Albuquerque et al., 2014). It is also possible that the cells introduced some drag on the signal as it traverses the device (Deng et al., 2019).

**FIGURE 5:**
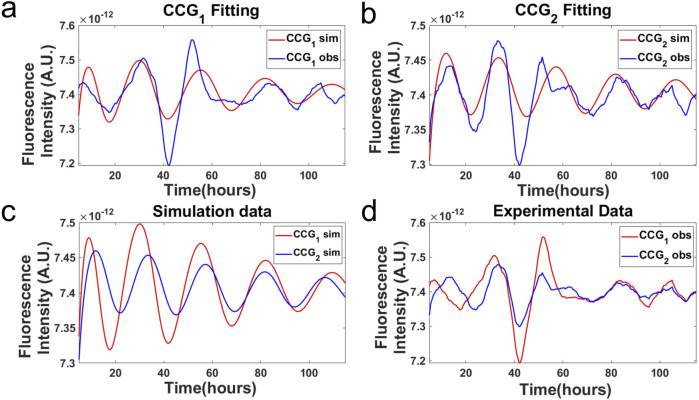
Trajectories of night owls and larks are plotted against each other and synchronized. The two populations of oscillators started 12 h out of phase and are labeled by a 1 or 2 to denote night owls and larks, respectively. Trajectories were also plotted against a quorum-sensing model identified by ensemble methods described below **(A,B)** (Cheong et al., 2022). Trajectories for both night owls (CCG1) and larks (CCG2) for a quorum sensing model are provided **(C)**. The observed trajectories for both night owls and larks are also given **(D)**.

The other microwell device acted to trap one or a few cells in 10 micron diameter wells, whose density can be varied from 2,000 to 16,000 wells (Figures 4e,f). The device is analogous to a microtiter plate. The top liquid layer over the wells acts as a possible medium for communication signals. The advantage of this device is that the fluorescent trajectories of individual cells can easily be measured over a 10 day window. In this way, the synchronization of individual trajectories over 5 days was observed (Figure 5).

In this device, a mixture of two populations of cells was created, one being 12 h out of phase with the other population. One population was a collection of night owls; the other was a collection of morning larks. The two populations were seen to converge in their oscillations, thereby seeing synchronization between the two populations of night owls and larks (Figure 5). The convergence is seen in both the data and the model for the clock, as described in Section 7.

While the study of conidia is quite tractable by microfluidics, the dominant life stage of a filamentous fungus is the filament. The same question of synchronization addressed for conidia can be asked about filaments but is complicated by the fact that the cell cycle and circadian rhythms may interact (Hong et al., 2014) to provide a new physical mechanism for synchronization through cell division (Cheong et al., 2024). Particular cell cycle genes may influence circadian rhythms (Hong et al., 2014), and others have argued that the coupling of cell cycle and circadian rhythms may result from generic gene replication events (Paijmans et al., 2015). To address the synchronization of growing filaments, it is necessary to design and construct a fourth microfluidics device with serpentine channels to enable tracking individual or a few filaments. By allowing the organism to grow, it is possible that a stronger circadian rhythm could be detected (Cheong et al., 2024).

The protocol for observing filaments in a serpentine device is given in Supplementary Figure S4. An agar block is placed at the edge of the device. The filaments grow into a chamber that is bordered by serpentine channels. As one or a few filaments enter a channel, they grow for 64 mm along a particular channel (Supplementary Figure S5) and are imaged. A time lapse video is given in Supplementary Figure S5 over 10 days.

As with the big chamber device, a measure of phase synchronization called the “Kuramoto K” was calculated between each pair of channels in the device (Table 2). The Kuramoto K is derived from each pair of instantaneous phase curves for a pair of channels. When K is near 1, the phase synchronization is nearly perfect. When the K is near 0, there is no phase synchronization. As an example, isolated cells from a droplet encapsulated device had a Kuramoto K near 0 (Caranica et al., 2019).

**TABLE 2:** Synchronization of pairs of serpentine channels measured by Kuramoto K (Cheong et al., 2024).

Kuramoto K	Serpentine 1	Serpentine 2	Serpentine 3	Serpentine 4	Serpentine 5
Serpentine 2	0.8272				
Serpentine 3	0.7937	0.7903			
Serpentine 4	0.7526	0.7866	0.7901		
Serpentine 5	0.8239	0.8763	0.8193	0.8106	
Serpentine 6	0.8148	0.8027	0.9045	0.8107	0.8511

What was seen in the serpentine device is that there is phase synchronization over long distances, raising the question: how does this happen? New physical models are needed to describe the physical processes underlying the clock and other complex traits (Cheong et al., 2024; Oyarte Galvez et al., 2025). In conclusion, we measured phase synchronization in both conidia and filaments. This phase synchronization potentially provides the basis for the emergence of a biological clock at the macroscopic limit of ∼150,000 cells and leaves open the mechanism for the phase synchronization of conidia and filaments.

## 6 Quorum-sensing (QS) signal/s

Having found that cells synchronize, the question is how they achieve this. Early theoretical work suggested the role of a shared signal in the medium, such as neurotransmitters, between cells in the suprachiasmatic nucleus of mammalian clock systems as a communication mechanism (Gonze et al., 2005). There are two lines of evidence (screens) for communication via quorum sensing in *N. crassa*. One comes from varying the density in a microwell device and measuring the fluorescence of larks and night owls over time. The second line of evidence comes from the development of continuous *in vivo* metabolism NMR (CIVM-NMR) to measure metabolites in real time in fungal filaments (Judge et al., 2019). CIVM-NMR provides the basis for a novel screen for quorum-sensing signal/s.

**FIGURE 6:**
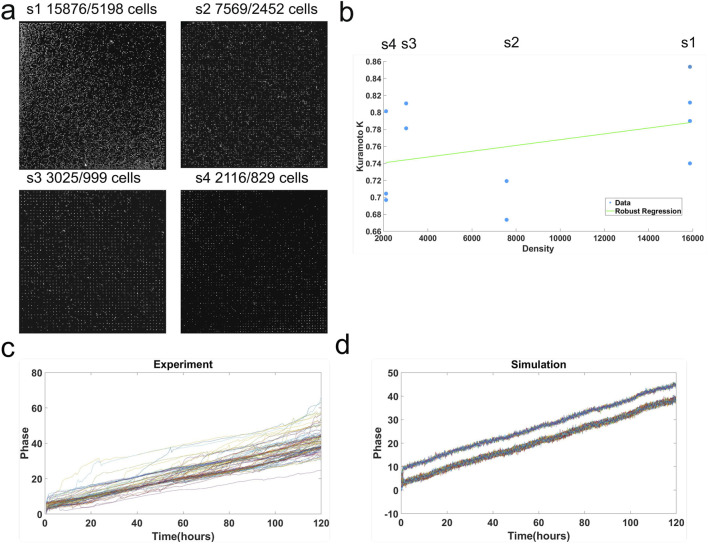
A microwell device is used to establish quorum sensing for the phase synchronization of single-cell clocks. **(a)** The microwell device supports densities of microwells from 2,000 to 16,000. **(b)** Phase synchronization measured by the Kuramoto K is density-dependent. **(c)** Instantaneous phase is plotted in the microwell experiment and **(d)** a fitted Kuramoto model to the same data (RHS) by ensemble methods (Cheong et al., 2022).

Using the microwell device, the microwells were populated with two populations of cells—night owls and morning larks—12 h out of phase. The microwell device contained microwell arrays at four densities. Fluorescent intensities were tracked in each well, and the Kuramoto K was calculated between the average trajectory of the night owls and morning larks at each density (Figure 6). There was a positive relationship between the Kuramoto K and density, indicative of quorum sensing. It is clear that CCG expression also varies with density.

As an additional check, a simple phase synchronization model known as the Kuramoto model (Shinomoto and Kuramoto, 1986) in statistical physics was also fitted to the data. The behavior of the Kuramoto model with its two populations of oscillators is shown in Figure 6 and compared with that from the instantaneous average phase of larks and night owls with similar response over time. The coupling constant was estimated to be over 10, and the measured phases were a little noisier as well (Figure 6c). The fitted model behavior supports the strong phase synchronization occurring between the oscillators.

**FIGURE 7:**
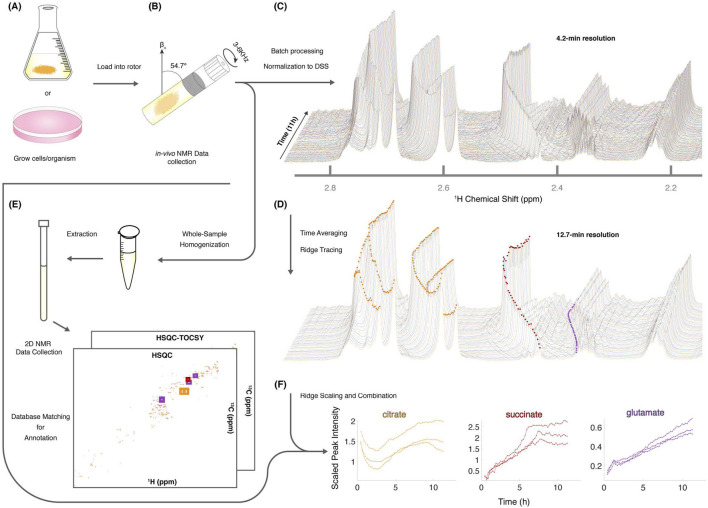
CIVM-NMR is used to carry out real-time measurement of metabolites of living filaments of *Neurospora crassa* **(A)**. **(B)** To remove broad NMR signals, a living sample of *N. crassa* is spun at the magic angle. **(C)** Batch processing and normalization by DSS leads to trajectories for over 400 1D features of metabolites at 4 min resolution. **(D)** Time-averaging and ridge-tracking (Wu et al., 2020) at 12 min resolution. **(E)** Living sample is removed and subject to HSQC and HSQC-TOCSY to extract 2D features for annotation. **(F)** Resulting annotation is used to examine replicate features of varied metabolites (Judge et al., 2019). Chemical Shift is measured in parts per million (ppm).

The second independent line of evidence for quorum sensing comes from metabolomics (Judge et al., 2019). In this approach, living filaments were placed inside a rotor in an NMR machine and spun at a magic angle (Figure 7). ^1^H NMR spectra were then collected in real-time at intervals of either 12 m or 4 m. By stacking the spectral features of metabolites, the metabolites were observed to evolve using CIVM-NMR (Wu et al., 2020; Wu et al., 2022).

This new method was applied to a classic mutant in *N. crassa* with unknown function, *qa-x*. This gene is part of a *qa* gene cluster metabolizing quinic acid (QA) which has been studied for over 60 years (Giles et al., 1985) as an early model for eukaryotic gene regulation. The only reported phenotype of *qa-x* was a darkening of the media (Supplementary Figure S6) (Judge, 2021).

A related mutant in human beings motivated Beadle and Tatum in their Nobel-Prize-winning work to develop a linkage between biochemistry and genetics (Beadle, 1958). A metabolic block arises in humans and leads to the accumulation of homogentisic acid (HGA) in the urine as well as aromatic amino acids, such as phenylalanine, tyrosine, and tryptophan. The motivating metabolic disease is alkaptonuria. This is precisely what the unknown *qa-x* mutant did metabolically in *N. crassa* (Giles et al., 1985; Judge, 2021).

**FIGURE 8:**
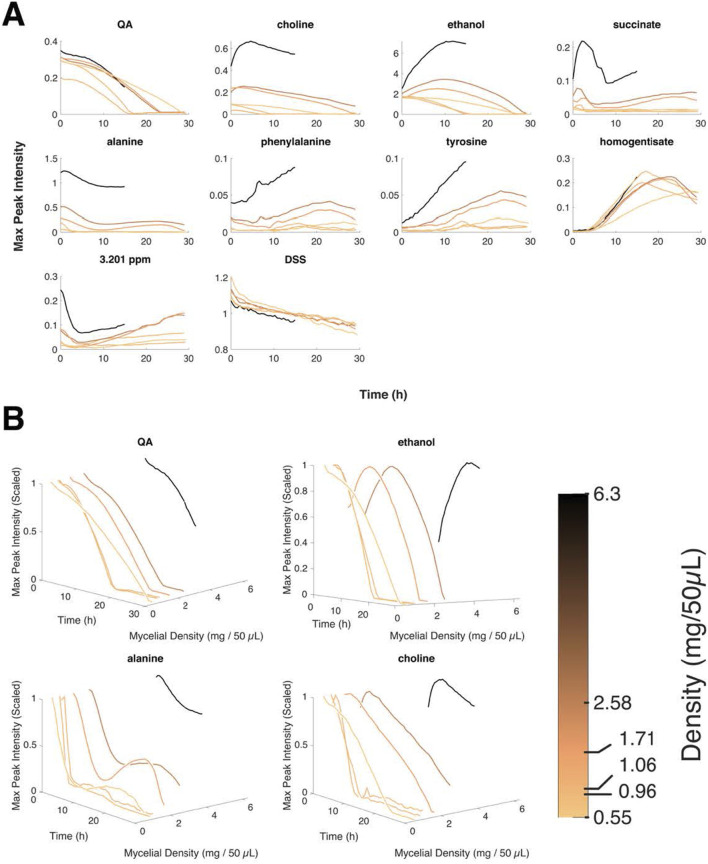
The *qa-x* mutant displays quorum sensing in ethanol production. **(A)** Varied metabolites are graphed as a function time. **(B)** Various metabolites are graphed as a function time and cell density. A sliding color bar encodes density for all metabolite curves (Judge, 2021). ppm is the chemical shift in parts per million, and 3.201 ppm is an unannotated feature. DSS is a NMR standard.

First, the media became discolored in *N. crassa* in this mutant background (like urine). The degree of accumulation of the oxidation products increased with the amount of QA in the media (Supplementary Figure S6C) (Judge, 2021). Then using CIVM-NMR, it was possible to identify the accumulation of HGA in living cultures along with the accumulation of tyrosine and phenylalanine (Figure 8) (Judge, 2021).

The surprise ending to the story connected to the motivating example of Beadle and Tatum’s work—alkaptonuria—is that under high density, ethanol was produced, and under low density ethanol was consumed (Figure 8) (Judge, 2021). The conclusion is that there is quorum sensing in the ethanol metabolism. The metabolomic response would suggest the aromatic alcohols as possible quorum-sensing signals (Chen et al., 2004).

What is not known at this stage is whether the quorum-sensing mechanism uncovered by the *qa-x* mutant is the same as in the clock (Cheong et al., 2022). In any case, metabolomics raises the possibility of increasing the utility of the Beadle and Tatum screen beyond genes in metabolism to uncover a variety of regulatory processes, including quorum-sensing mechanisms in *N. crassa*. These density-dependent effects may have other effects in addition to the clock in, for example, conidiophore architecture (Krach et al., 2022).

The exploration of circadian rhythms through metabolomics is likely to uncover new mechanisms by which metabolism feeds back on the clock independently of the core oscillator mechanism involving the transcription–translation feedback loops in Figure 1A. Wulund and Reddy (2015) have hypothesized that the redox state of the cell may provide the feedback necessary to establish circadian rhythms even when the core oscillator is not available. This feedback of the Redox state may also help explain the between linkage the clock and aging (Judge et al., 2017).

## 7 Communication mechanisms

In the spirit of a strong inference framework (Platt, 1964), one of two very different communication mechanisms were considered to be at work in a clock network (Supplementary Figure S7). The goal was to test quorum sensing against another hypothesis: contact communication (Nudleman et al., 2005).

One mechanism involves quorum sensing (Supplementary Figure S7a). Under this hypothesis, the quorum-sensing signal is produced by a *clock-controlled gene’s* protein product, CCG. The CCG level with its mCherry recorder provides the fluorescent trajectory associated with the expression of a well-studied CCG-2 promoter (Castro-Longoria et al., 2010). Once the quorum-sensing signal is produced, it is transported to the media. Then all the cells producing the quorum-sensing signal and exchanging it with the media can set their clocks to the level of the signal in the medium. This will lead to phase synchronization.

This hypothesis might explain the fluorescent behavior of *N. crassa* cells in a microwell device that includes the classic mixing of two cell populations out of phase. In Bier et al. (2000), two *Saccharomyces cerevisiae* populations out of phase for their glycolytic oscillations synchronized their oscillations (Figure 5). We carried out a similar mixing experiment with two *N. crassa* cell populations 12 h out of phase to examine the phase synchronization of their circadian rhythms. An example of how quorum sensing leads to phase synchronization in a microwell device is shown from a model fitted by ensemble methods to data on a microwell device (Deng et al., 2019).

Several specific assumptions were made in the quorum-sensing model. The first is a mean field assumption (Gonze et al., 2005) about the quorum-sensing signal S_e_ diffusing instantaneously and uniformly throughout the device. The evidence for this comes from the big chamber device (Figure 4A). The diffusion assumption has been successfully used in modeling the syncytium of the *Drosophila* developing blastoderm (Jaeger et al., 2004). The last major assumption is the way in which the quorum-sensing signal interacts with the clock mechanism. Deng et al. (2016) found that a reasonable way in which the quorum-sensing signal interacted with the clock mechanism was in a negative effect on WCC. In this way, WCC is viewed as the receiver for the quorum-sensing signal as WCC is for light (Froehlich et al., 2002).

An alternative hypothesis to quorum sensing is a contact model of communication between cells (Supplementary Figure S7b). An example of this form of communication is found in *Myxococcus xanthus* (Nudleman et al., 2005) and possibly *Anabaena* filaments (Arbel-Goren et al., 2021). Most of the same assumptions are in place, except that under the contact hypothesis, cells communicate through a signal by cell-to-cell contact (Supplementary Figure S7b). The communication signal can only be exchanged with another cell in physical contact.

Both models (Supplementary Figure S7) were fitted by ensemble methods to fluorescent data from a mixed population in a microwell device composed of larks and night owls observed over 100 h or more. Quorum-sensing models were fitted by ensemble methods (Cheong et al., 2022) described in the last section of this review (Figure 5b). The larks and night owls were initially 12 h out of phase. An ensemble method presumes that the data are limited, but there are many unknown parameters in the genetic network. An average is taken over the ensemble of 40,000 models to make predictions over time of the conidial cell fluorescence. It can be seen that the larks and night owls are quite well predicted by the quorum-sensing model, as their respective oscillators come into phase (Figure 5). The minimum $\chi^{2}=2016$ with 442 fluorescent time points is shown in Figure 5.

The same ensemble method was applied to the contact model fitted to the same data set. Goodness-of-fit was measured by the minimum $\chi^{2}=4373$ (Supplementary Figure S8a)—considerably worse than the quorum-sensing model. The failure in fit appeared to be due to finding a model in which the communication signal had damped oscillations (Supplementary Figure S8d). The fitted ensemble was unable to track the CCG-2 fluorescence data (Supplementary Figure S8c). We concluded that the contact model failed to explain the fluorescence data from the microwell device (Cheong et al., 2022).

## 8 Stochastic resonance (SR) as physical hypothesis

Stochastic resonance (SR) is a physical theory about how noise at an intermediate level can amplify a desired signal; it was first introduced as a physical hypothesis by Benzi et al. (1981) in the study of climate change. Since its introduction, it has been widely applied in engineering to amplify a signal in the presence of noise (Jiao et al., 2019). For example, if the desire was to listen to a symphony on public radio, the quality of the signal could be improved by adding the right amount of white noise across the spectrum. This may at first seem entirely counter-intuitive, but there are even more surprising effects of noise on oscillatory systems (Saxena and Kosterlitz, 2019).

A simple rationale of how SR leads to oscillations was provided by Rappel and Strogatz (1994) and described in Section 3. In this scenario, noise is the driving agent in producing circadian rhythms when there is an intermediate level of noise in the genetic network, as it does in other aspects of gene function (Cai et al., 2008; Lin et al., 2015).

Sriram and Gopinathan (2005) hypothesized that SR could explain circadian rhythms in *N. crassa*. There was then no evidence for the validity of this hypothesis. Microfluidics had yet to be applied in *Neurospora* to measure single-cell behavior at the time this hypothesis was made. The ideal domain for the application of this hypothesis remains circadian rhythms in single microbial cells.

The importance of SR arises in the context of single cells, in which stochastic intracellular noise can have an impact on the clock in single cells. In this context, the species in the genetic network are no longer concentrations but are actual counts of molecules such as RNAs and proteins (Caranica et al., 2020). In such a setting, there is noise in each species within the cell in the network. The model is a full stochastic network in which each species is counted within a cell and is noisy (Gillespie, 1977). The counts of molecules in cells are thus intrinsically noisy (Elowitz et al., 2002). Genes exist in one or few copies and their RNA and protein products are amplified by transcription and translation to tens or maybe hundreds of copies within a single cell.

The process of identifying this stochastic network is now described briefly (Caranica et al., 2018; Caranica et al., 2020). Noisy trajectories in individual cells were captured in a droplet encapsulating device as an example (Figure 3). These trajectories were transformed into the frequency domain to produce a power spectrum for each cell. Particle swarm optimization methods were then used to fit the stochastic network to the power spectrum averaged across cells (see last section on ensemble methods) (Thomas et al., 2013; Caranica et al., 2018; Caranica et al., 2020). In carrying out this fitting process, both the experimental errors and stochastic intrinsic error were separated and accounted for (Deng et al., 2016; Caranica et al., 2018). Finally, a method of measuring the noise in the cell was used to explore the effects of the stochastic intracellular noise on the oscillations (signal) in the stochastic network to examine SR as a function of the stochastic intracellular noise.

**FIGURE 9:**
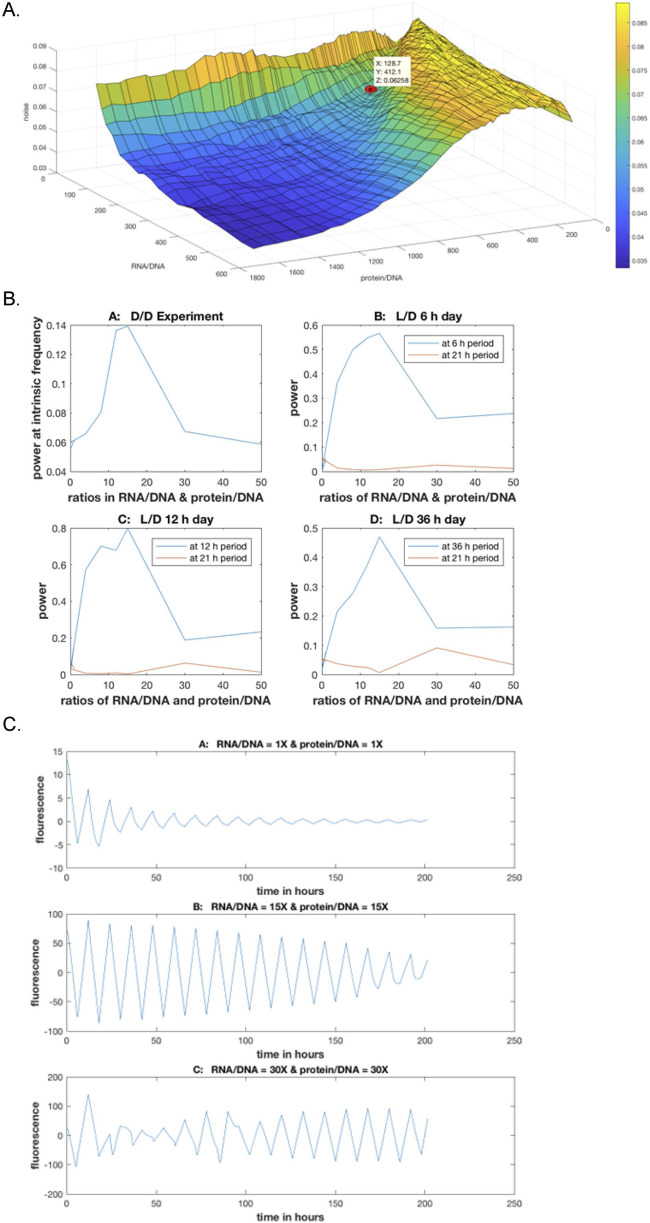
**(A)** Stochastic intracellular noise is a function of the RNA/DNA and protein/DNA ratios in the cell and is determined experimentally. Red dot is the measured value for *N. crassa* (Caranica et al., 2020). **(B)** Signal provided by the maximum in the power spectrum shows a stochastic resonance (SR) at an RNA/DNA or protein/DNA ratio of approximately 15 across varied daylengths (Caranica et al., 2020). **(C)** SR in circadian rhythms displayed in single-cell trajectories as the stochastic intracellular noise is varied through the ratios of RNA/DNA and protein/DNA in *N. crassa* (Caranica et al., 2020).

What traditionally distinguishes stochastic networks from deterministic genetic networks is a size parameter for the cell that controls the noise in the cell and is missing from deterministic models (Wu et al., 2011). We developed an experimental means to identify the size parameter in stochastic networks and varied it (in the fitted models) without varying the rate coefficients (Figure 9A). Stochastic intracellular noise was quantified from the measured total RNA/DNA and protein/DNA ratios, thus amplifying genes into their concomitant products. When there is more amplification, there is less noise in the system (Figure 9A). The measured point of the system is indicated on the surface (in red), and the surface as a whole was generated from the fitted ensemble. A total of 1,024 stochastic trajectories, or Gillespie trajectories (Gillespie, 1977), were generated at each grid point defining the noise surface. Then the location on the surface was used to determine whether or not there was SR (Caranica et al., 2020).

The power (signal) was measured as a function of the same ratios in RNA and protein amplification (Figure 9B) (Caranica et al., 2020). The power spectrum in the dark (D/D) served as a control as the day length is varied. The striking observation was that the SR for all light entrainment responses occurred at an amplification ratio of ∼15 in RNA and protein. There is a common SR independent of day length experienced by the organism, including that of a D/D run totally in the dark.

The direct outcome on fluorescent trajectories by the stochastic intracellular noise can also be examined (Figure 9c) (Caranica et al., 2020). When the noise is too low or high, there is degradation in the circadian rhythms. At the SR, there was a strong circadian rhythm. In addition, a second defining property, a biological clock, is entrained with a light or temperature signal (Merrow et al., 2006). As shown here, the same SR is evident as the clock entrains to a 6, 12, or 36 h day.

What is needed now is to manipulate the stochastic intracellular noise directly in the experimental system in a microfluidics device.

## 9 Stochastic coherence (SC) and transcriptional bursting

Stochastic coherence (SC) is another physical hypothesis that also explains the emergence of the biological clock from the behavior of clocks in single cells. Under this hypothesis, the clock network only has two species that are stochastic: the gene encoding the oscillator *frq* and the gene *ccg* encoding the protein that makes the quorum-sensing signal. The only stochastic part of the model is the random flipping of these two genes on–off or off–on. This random flipping in the state of each gene is known as “transcriptional bursting” (Zoller et al., 2018). Key parameters of this model are the rate coefficients of the activation and deactivation of the clock genes (Nicolas et al., 2018).

The model is summarized in Supplementary Figure S9. The model is almost entirely deterministic. Only *frq* ^0^, *frq* ^1^, *ccg* ^0^, and *ccg* ^1^ are random. The rest of the species follow the deterministic dynamics in Figure 1A. In contrast to SR with flipping between equilibrium states due to noise, this SC hypothesis has the model flipping between four deterministic dynamics. The model is referred to as a “hybrid model” because it is intermediate between the full stochastic model of the previous section and the deterministic model in Figure 1A (Wu et al., 2024). The dynamics of the hybrid model capture most single-cell cellular dynamics of the full stochastic model using ensemble methods (Figure 1A) (Deng et al., 2019). Even though the full stochastic model has all species random, the hybrid model explains the average power spectrum of single cells almost as well as the full stochastic network (Deng et al., 2019).

The hybrid model has rich dynamics. An examination of the dynamics of WCC (Supplementary Figure S10A) reveals that while individual cells are quite erratic in maintaining a 21 h period in one part of the parameter space, the average of the 500 cells with the network above flipping on and off has very regular behavior in WCC. Individual trajectories appear to engage in beat skipping, but there is no such behavior in the average WCC trajectory over the 500 oscillators communicating by quorum sensing. If one examines the power spectrum of individuals cells in the 500-cell collection, very little structure is apparent. In contrast, the power spectrum of the average trajectory of 500 cells shows structure.

The hybrid model is rich in another behavior, being in lock-step between trajectories. In other parts of the parameter space, there is no beat skipping (Supplementary Figure S10B). Lock-step behavior between trajectories of different cells is observed. What is also interesting is that when the quorum-sensing signal is removed, WCC shows little clock behavior. Oscillations of the average trajectory were small and damped out. The same behavior was shown when quorum is removed in a model with beat skipping.

In fact, consideration of the simpler hybrid model established that necessary and sufficient conditions for circadian rhythms to emerge were: 1. the presence of a quorum sensing signal and 2. transcriptional bursting occurring in the oscillator encoding gene *frq* and quorum-sensing encoding gene *ccg*.

**FIGURE 10:**
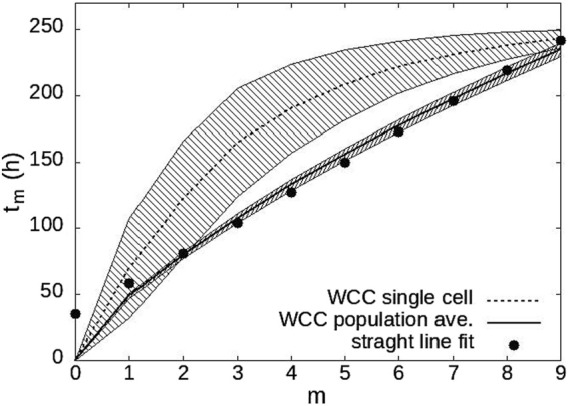
The biological clock is an emergent property of 500 cellular oscillators. Spacing between maxima is on the y-axis. Index of the maximum along a trajectory is given on the x-axis. A strong linear relationship emerges for the population average of WCC over the 500 oscillators displays a well-defined linear clock (Wu et al., 2024).

The final feature of the model was showing how the biological clock emerged from the phase synchronization of individual cellular oscillators (Figure 10). If the individual WCC trajectories are used to clock the time, the clock time of the maxima was nonlinear as a function of the ascending integer index of the maxima in the trajectories. If instead the average trajectory of WCC over 500 conidia was used, there was a nearly perfect linear relation between the time and index of the maxima.

**FIGURE 11:**
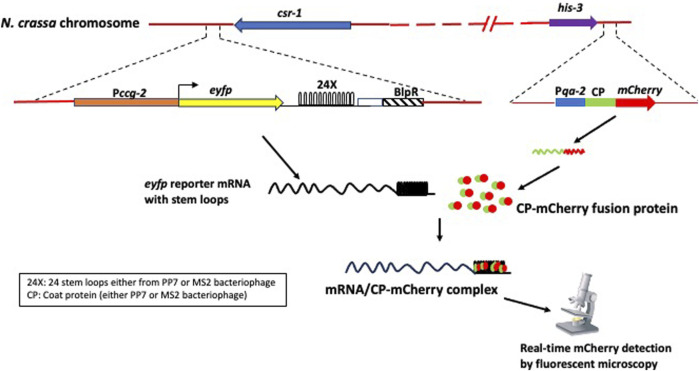
Strategy for measuring transcriptional bursting in the stochastic coherence hypothesis (“hybrid model”) with a new genetic construct.

A key experimental test of this model will be to measure transcriptional bursting (Figure 11). These experiments are under way. The approach is to detect a fluorescently labeled reporter mRNA in real time by fluorescent microscopy in *N. crassa*. The reporter mRNA containing an array of either PP7 or MS2 bacteriophage-specific 24 tandem stem loops downstream of the coding sequences will be expressed using the Pccg-2 promoter from the *csr-1* locus (Castro-Longoria et al., 2010; Deng et al., 2019). A fluorescently labeled (mCherry) either PP7- or MS2-specific capsid protein will be expressed from the *his-3* locus. The binding of fluorescently labeled capsid protein to the stem loops allows visualization of single mRNAs (Kaganman, 2008; Vera et al., 2016; Tutucci et al., 2017). Transcriptional bursting can thus be measured directly, and single mRNAs can be localized within a filament in real time. This approach will provide a direct test of the SC hypothesis as well as a platform for screening transcriptional bursting in other clock mechanism genes.

Consequently, under the SC hypothesis, it has been shown how the biological clock emerges from single-cell behavior, and SC is tractable to testing by visualizing single clock mRNAs. The model is also more tractable to analysis than the full stochastic model. Finally, it captures most of the variation in single-cell fluorescence relative to the full stochastic model (Deng et al., 2019).

## 10 Ensemble methods for fitting genetic network models from statistical physics

Ensemble methods were originally developed by Boltzmann for problems in statistical physics (Guggenheim, 1955) but now have been applied in a number of areas outside of physics (Landau et al., 2014). Consider the motion of an ideal gas in a 1 L volume. There are in the order of 6 × 10^23^ molecules each with three coordinates and three momenta. The challenge is to describe their motion when only pressure (P), volume (V), and temperature (T) are measured. The degrees of freedom vastly outnumber the number of measurements. To overcome this problem, Boltzmann suggested giving up on determining the best model for the system but instead advocated calculating the average behavior of the system instead from an ensemble of models consistent with the data available.

Ensemble methods were first introduced into systems biology to describe the behavior of a genetic network for the *qa* gene cluster (Battogtokh et al., 2002), and then the behavior of the clock network, in *N. crassa* (Figure 1A) (Yu et al., 2007). While the problems of having many parameters and limited data are not as severe as for an ideal gas, in the context of genetic networks there are still many unknown parameters and limited data available at the molecular level. Only a few species can usually be measured at different times, while there are many species in the genetic network. The goal of ensemble methods is to reconstruct the likelihood function by Monte Carlo (Landau and Binder, 2009) and then to average over the resulting ensemble of models consistent with the data available to predict systems behavior over time. Ensemble methods tell us both what we know and also what features of the model are not well specified by the data. The ensemble makes suggestions on how to improve the model (Dong et al., 2008) in successive rounds of experimentation with networks. This approach has been highly successful for a variety of networks (Chakraborty and Das, 2010).

**FIGURE 12:**
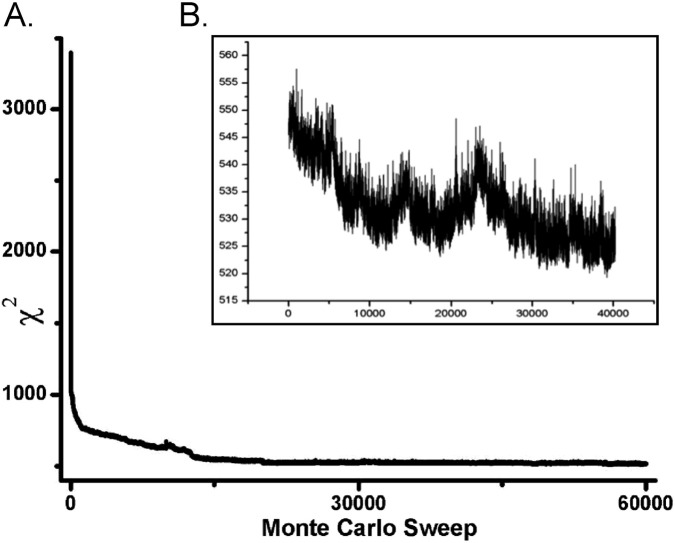
**(A)** Equilibration run to determine the clock network in Figure 1A. On the y-axis is the χ^2^ goodness-of-fit criterion plotted against sweep (one visit, on average, to each parameter) (Yu et al., 2007). **(B)** Blow-up of bump in equilibration in **(A)** to show that the Monte Carlo reconstruction of the likelihood is not a greedy search. The χ^2^ goodness-of-fit criterion is on the y-axis; sweep is on the x-axis. A sweep is a visit to each parameter once on average.

One of the earliest applications of ensemble methods in systems biology was to the clock network on a macroscopic scale (Figure 1A) (Yu et al., 2007). The ensemble was reconstructed by Monte Carlo methods. Each parameter was twiddled on average once per sweep. When the twiddled parameter improved the model fit, the twiddle was accepted. If the twiddle led to a decrease in fit, the twiddle was occasionally accepted as well. Accepting moves in the parameter space that decreased the likelihood of the parameter choice or, equivalently, increased the goodness-of-fit measure, such as the χ^2^, allowed escape from local optima. The search phase is called “equilibration.” An example of this process is shown in Figure 12A. A blow-up of one region (Figure 12B) displays the up-and-down movement of the Monte Carlo search for a good set of parameters consistent with the data as measured by the χ^2^ goodness-of-fit measure. The up-and-down movement is an indication that the search for a good set of parameters is not greedy. Typically, this goodness-of-fit improves and stabilizes during the equilibration phase.

**FIGURE 13:**
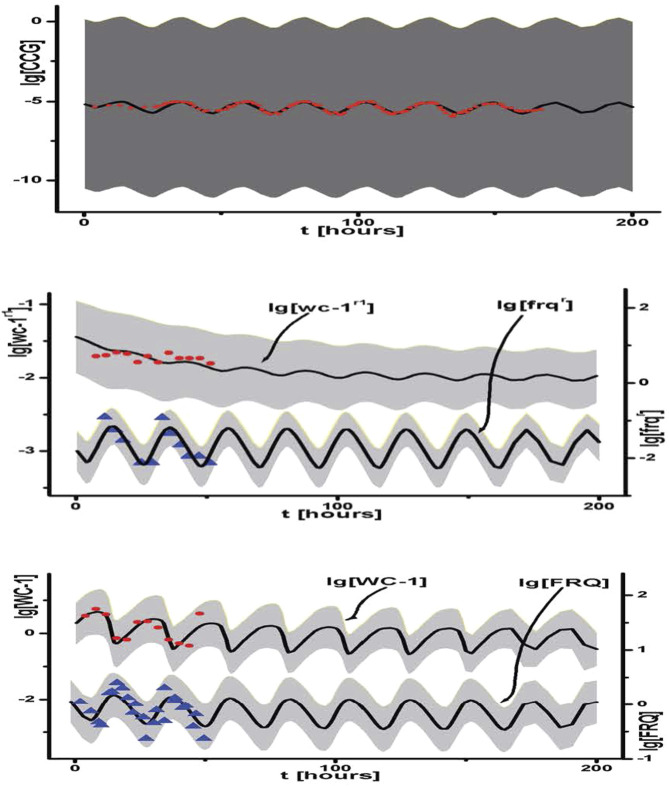
Ensemble for Figure 1A fitted to molecular and physiological data on clock (Yu et al., 2007). These same ensemble methods were then subsequently applied to single-cell data to examine synchronization hypotheses (Figure 5) (Cheong et al., 2022).

Once the Monte Carlo run equilibrated after typically 40,000 sweeps, then an accumulation phase is begun in which 40,000 models are accumulated to reconstruct the likelihood function. Averaging over the 40,000 models in the accumulation phase is used to predict model behavior over time and assess fit to the data (Figure 13). The model ensemble predicts very well the behavior of race tube data on the clock and its physiological output. In addition, molecular species such as the mRNAs and polypeptides of *wc-1* and *frq* are well also predicted. This genetic network is relatively small, with only 26 rate constants and 16 initial conditions for the molecular species. The shaded areas are predictions plus or minus two error bars on the predictions of different species. Similar methods are used to predict the paths of hurricanes by ensemble methods.

The same methods were recently applied at the microscopic, single-cell level to stochastic networks (Caranica et al., 2018; Caranica et al., 2020). Again, these models were challenging to fit. To make this problem tractable, the power spectrum in the frequency domain was used to guide the estimation of the model ensemble. The model ensemble captures two of three critical statistics: amplitude and period of an oscillator. Special new Monte Carlo methods were developed for stochastic networks to address this fitting problem, called “particle swarm optimization methods” (Caranica et al., 2020). The remaining property of an oscillator—phase—was then used as an independent piece of the trajectory to assess goodness-of-fit (Caranica et al., 2020).

**FIGURE 14:**
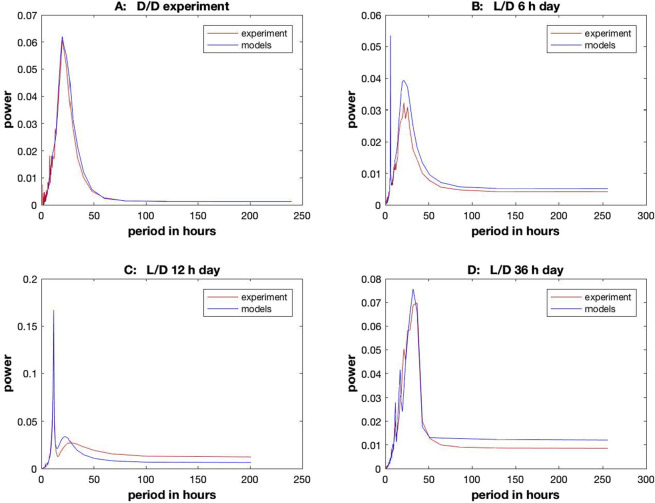
Ensemble of stochastic networks fitted to encapsulated microfluidics data on single cells (Caranica et al., 2020) for in the dark DD **(A)** and for a Light/Dark (L/D) cycle for a 6 hour day **(B)**, 12 h day **(C)**, and 36 h day **(D)**.

The results of this ensemble method are shown in Figure 14. The day length was varied from experiments in the dark (D/D) to several D/L entrainment experiments. In all cases, the resulting ensemble predicted the power spectra quite well. Similar methods were also successfully applied to the hybrid model (Deng et al., 2019).

There are three challenges to fitting genetic networks to macroscopic or microscopic data in systems biology. The first is overcoming the problem of many parameters (p) and limited data (N << p). This challenge is overcome by ensemble methods from statistical physics (Landau et al., 2014).

The second challenge is that networks tend to be quite large. This has been overcome by: i. developing new parallel algorithms for solving ordinary differential equations (Al-Omari et al., 2013); ii. developing new parallel algorithms on GPUs; and iii. using multiple search agents on the parameter space, such as particle swarm optimization (Caranica et al., 2020). All these approaches are successful as part of ensemble methods for large networks. The last problem is identifying a genetic network of unknown topology. Again, this problem has also been successfully addressed using variable topology ensemble methods (Al-Omari et al., 2022).

**FIGURE 15:**
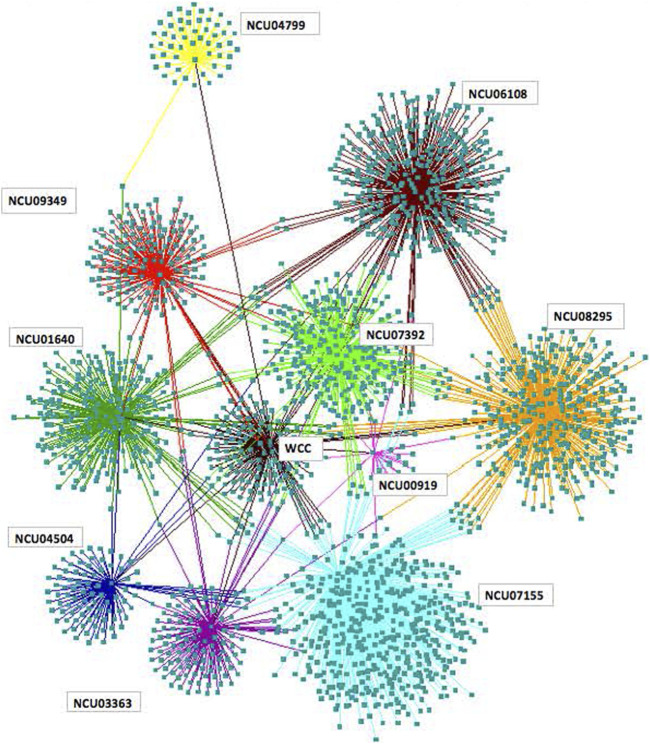
Clock regulatory network with 3,380 genes visualized using Cytoscape (Shannon et al., 2003). Each ball captures the targets of a transcription factor or RNA-binding protein defining an RNA operon. Five positive transcriptional regulons and six RNA operons are shown. The *Neurospora crassa* identification number (NCU) is given for each regulator (Al-Omari et al., 2022).

The result of addressing all three of these problems is the ability to successfully identify an ensemble of models for the whole clock network (Figure 15) (Al-Omari et al., 2022). There are 3,380 genes in the clock network and at least 11 regulators. Five of these positive regulators are transcriptional; six are RNA operons. This network is comparable in size to the whole *E. coli* transcriptional network and links metabolism with gene regulation. One of the striking features of this network is that the RNA operon controlled by LHP-1 (NCU08295) is larger than the DNA regulon of WCC. The role of post-transcriptional regulation is not to be under-estimated in the clock network.

A basic issue about such large networks is identifying their building blocks (Milo et al., 2002). The analogy is to the resistors, capacitors, and other components of electrical circuits. This problem of identifying network motifs represents a fundamentally new combinatorics problem in mathematics. One analytical solution is available for feedforward loops (Al-Omari et al., 2022) and is illustrated in the clock network. A total of 71 feedforward loops were identified as significant in the clock network. This is larger than the number in the *E. coli* transcriptional network (Milo et al., 2002). Among 71 feedforward loops, 39 were associated with the *lhp-1* and NC6018 regulator genes, both of which are associated with ribosomes and ribosome biogenesis. New tools are now becoming available in mathematics to identify such building blocks in large genetic networks (Scruse et al., 2024).

Such large networks where the number of parameters exceeds the number of data points also provide particular challenges for the design of future omics experiments to identify the ensembles describing the clock. Classical experimental design only covers the situation where the number of data points exceeds the number of parameters (Fisher, 1935; John, 1971; Ryan and Morgan, 2007). New ensemble approaches for the adaptive design of omics experiments for the discovery of ensembles that describe the clock network are only now being developed (McGee and Buzzard, 2018; Torres et al., 2025).

The initial screen based on transcriptional profiles of RNA-binding proteins and transcription factors to flush out the entire clock network has been recently improved with the use of luciferase recorders on 289 transcription factors (Muñoz-Guzmán et al., 2021). It would be very interesting to see how the network (Figure 15) changes with the new hubs added (Muñoz-Guzmán et al., 2021).

## 11 Future directions

Table 1 lays out four different hypotheses about how a collection of oscillators might synchronize with or without stochasticity in the cell and with and without the communication mechanism quorum sensing between cellular oscillators. In the absence of quorum sensing, experimentally in a droplet encapsulating microfluidic device there is no synchronization of oscillators in different droplets. Using a large chamber microfluidic device, it is demonstrated that the macroscopic limit in an artificial tissue of ∼150,000 conidia is achieved, in which two cycles of the clock are maintained over 48 h, as seen in liquid cultures with 10^7^ cells. In a microwell device, it is also shown that the Kuramoto K measure of phase synchronization is density-dependent on conidia and accompanied by changes in expression at the *ccg-2P* promoter at the single-cell level (as hands on the clock). Thus by definition, quorum sensing is involved in phase synchronization of single-cell clocks. Independently using CIVM-NMR on living filaments, it has been shown that the production of ethanol displays quorum sensing. It is not known whether the two quorum-sensing mechanisms are the same or different. Are there multiple quorum-sensing signals at work in the clock? Are there new NMR methodologies, such as CIVM-NMR (Judge et al., 2019), that allow us to screen now for quorum-sensing signals associated with particular proteins in much the same way that Beadle and Tatum screened for genes connected with particular proteins?

The physical hypothesis of stochastic resonance, in which noise plays a positive role in circadian oscillations, is partially validated by fitting new stochastic networks to single conidial data in droplet encapsulating microfluidic devices. The fitting of stochastic networks to single-cell data involved the development of new ensemble methods utilizing particle swarm optimization algorithms to identify model ensembles. The stochastic internal noise of the cell is quantified by the use of total RNA/DNA and protein/DNA ratios in the conidial cells. As these ratios are varied in the same way without changing the rate constants in the clock network, a clear stochastic resonance emerges and manifests itself in the oscillations seen in fitted individual single-cell trajectories. What is missing is an experimental manipulation of the stochastic intracellular noise to validate the stochastic resonance in *N. crassa* (Elowitz et al., 2002). Just as the level of noise in the random directions of each fish or bird matters in transitioning to coherent motion, so does the level of stochastic intracellular noise in seeing the emergence of the phase synchronization of cellular clocks.

A second physical hypothesis that represents a compromise between the full stochastic model underlying the stochastic resonance hypothesis and the deterministic quorum-sensing model developed for microwell devices was tested and found to be almost as successful in explaining single-cell data as the full stochastic model. This hybrid model is called the “stochastic coherence hypothesis” to emphasize both its similarity to stochastic resonance as well as its difference. There are two elements to this model: 1. quorum sensing; 2. random flipping on and off of the *frq* and *ccg* genes producing the quorum-sensing signal—transcriptional bursting. In stochastic resonance, the mechanism for obtaining oscillations pushes the system from one fixed point to another to achieve oscillations; in stochastic coherence, the dynamics are randomly flipped between four deterministic clock networks to achieve oscillations. In stochastic coherence, the random flipping of the *frq* gene is the central, robust driving agent of the clock oscillation, and not merely an auxiliary or a noisy disturbance of the clock. It is shown that quorum sensing and random gene flips in the hybrid model are necessary and sufficient to generate a biological clock for a population of 500 or more oscillators. What is unknown at this time is the level of noise needed to ensure that a population of cellular oscillators can produce a population clock and whether there is, additionally, stochastic resonance at work, as in Figure 12, that contributes to the emergence of the biological clock. We have developed new tools for measuring transcriptional bursting in the *N. crassa* clock utilizing the MS2 and PP7 capsid protein systems for measuring single RNAs to observe the bursting.

There is much to be discovered experimentally and theoretically about the driving role of transcriptional bursting in creating circadian systems as well as the factors, such as quorum, in coupling a population of circadian cellular oscillators to produce a clock. New experimental and theoretical approaches from metabolomics, microfluidics, and statistical physics will be needed to enable these discoveries.

## Supplementary material

SUPPLEMENTARY FIGURE S1 Left panel **(A)** is time lapse growth in a race tube of a *band* (*bd*) mutant. Right panel **(B)** is time lapse video of *ccg-2* luminescence (Gooch et al., 2008) in a liquid culture. See Supplementary Videos S2, S3.

SUPPLEMENTARY FIGURE S2 Droplet encapsulation from the encapsulating microfluidic device (Deng et al., 2016). Fluorescent beads are captured in each droplet. See Supplementary Video S4. Two frames of the video are shown on left **(A)** and right **(B)**.

SUPPLEMENTARY FIGURE S3 Pairwise synchronization between five fields of view in an artificial tissue created within a large chamber device. On the Y-axis is the instantaneous phase of one field of view plotted alongside the instantaneous phase of another field of view. On the X-axis is time in hours. The phase pairs of all ten pairs of fields of view are plotted against time (Cheong et al., 2022).

SUPPLEMENTARY FIGURE S4 A serpentine device is used to support filament growth in serpentine channels. Chips are imaged in a temperature- and light-controlled environment (Cheong et al., 2024).

SUPPLEMENTARY FIGURE S5 Growth of filaments in channels of serpentine device over 200 h. See Supplementary Video S5. The dimensions are 65,500 × 20,150 μm . Typically, the length of two U-turns is 1,312 μm and the total serpentine channel is 198,241 μm . Each device has 95 serpentinue channels.

SUPPLEMENTARY FIGURE S6 Cultures of the *qa-x* mutant accumulate pigmented compounds in the media. **(A)** Comparison with wild-type (OR74A). **(B)** Media alone. **(C)** Titration of pigmented compounds by varying quinic acid (QA) in media (Judge, 2021).

SUPPLEMENTARY FIGURE S7
**(a)** Quorum-sensing model and **(b)** contact model (Cheong et al., 2022).

SUPPLEMENTARY FIGURE S8 Contact models fitted to single-cell data in microwell data as in Supplementary Figure S3B. **(a)** Ensemble of contact models was equilibrated. **(b)** Critical communication rate parameters were equilibrated. **(c)** One cluster of oscillators was fitted to the single-cell data. **(d)** The communication signal molecule in the two giant cells from night owls and larks damped out. (Cheong et al., 2022).

SUPPLEMENTARY FIGURE S9 Hybrid model with transcriptional bursting *frq* and *ccg* (Wu et al., 2024).

SUPPLEMENTARY FIGURE S10
**(A)** Averaging over 500 oscillators with quorum sensing and transcriptional bursting produces regular clocklike behavior in WCC. Individual trajectories (with arrows) can skip a beat or two. The power spectrum of the average trajectory has structure, but the average of the power spectra of individual cellular oscillators lacked structure (Wu et al., 2024). **(B)** Some regions of the parameter space of the hybrid model displayed no beat skipping, but individual trajectories are in lock step (Wu et al., 2024).
