# Supplementary figures and images for: Origin of the clock in Neurospora crassa
Source: Front Mol Biosci. 2026 Jan 14;12:1697003. doi: 10.3389/fmolb.2025.1697003 (PMC12848316; doi:10.3389/fmolb.2025.1697003)

A

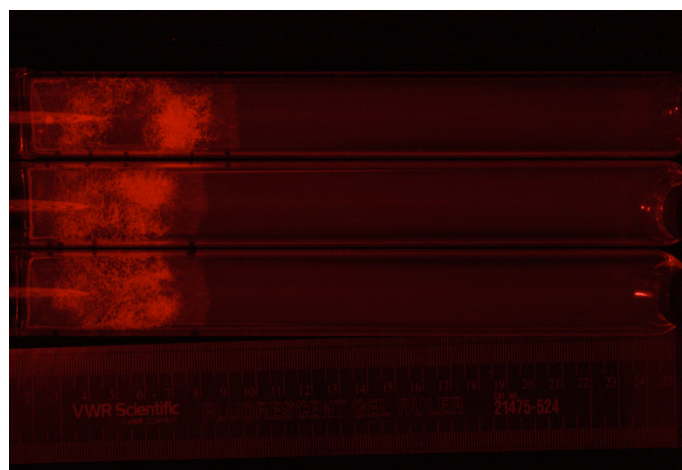

B

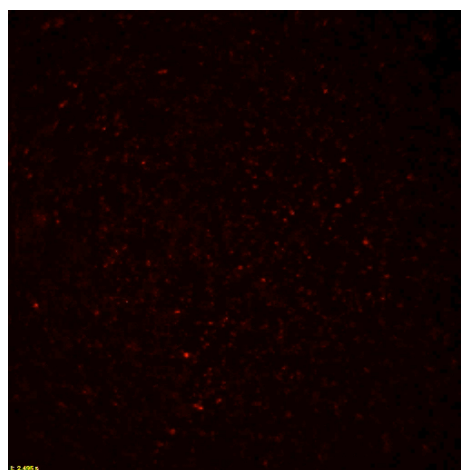

Supplement: Supplementary file 1 [file Supplementaryfile1.zip › Supplementary File 1/Supplementary Figure 1.pdf]

A.

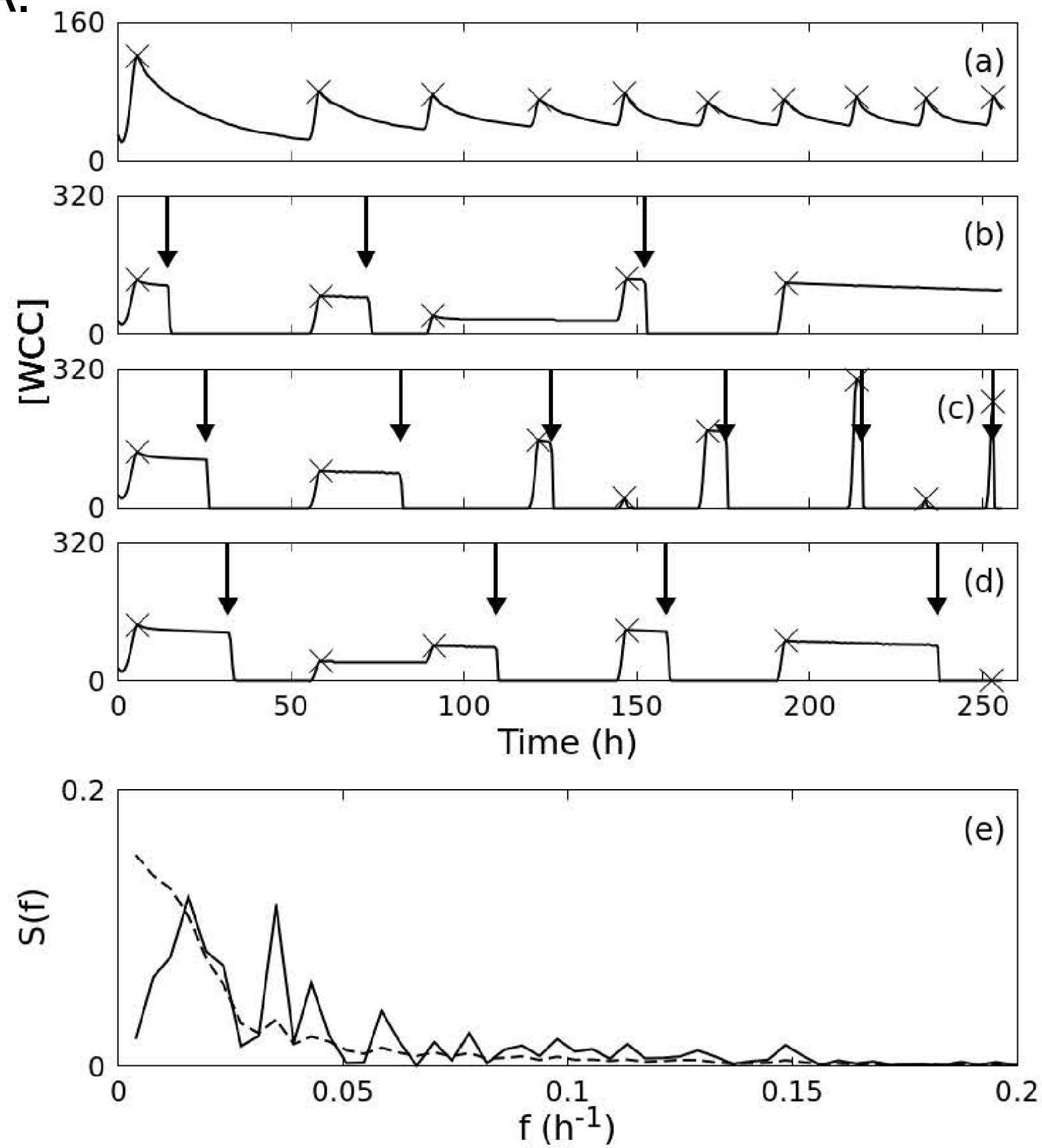

B.

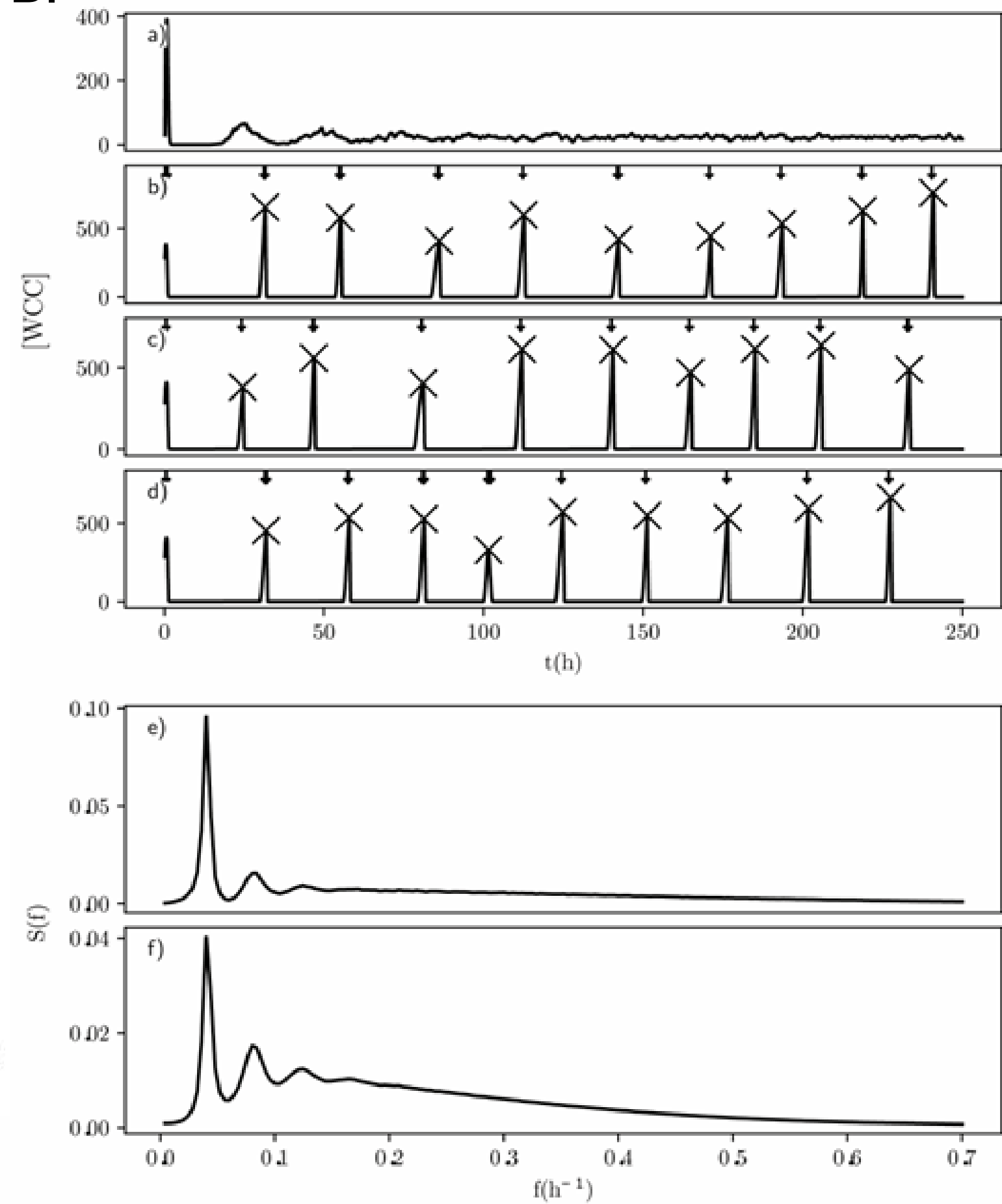

Supplement: Supplementary file 1 [file Supplementaryfile1.zip › Supplementary File 1/Supplementary Figure 10.pdf]

**A**

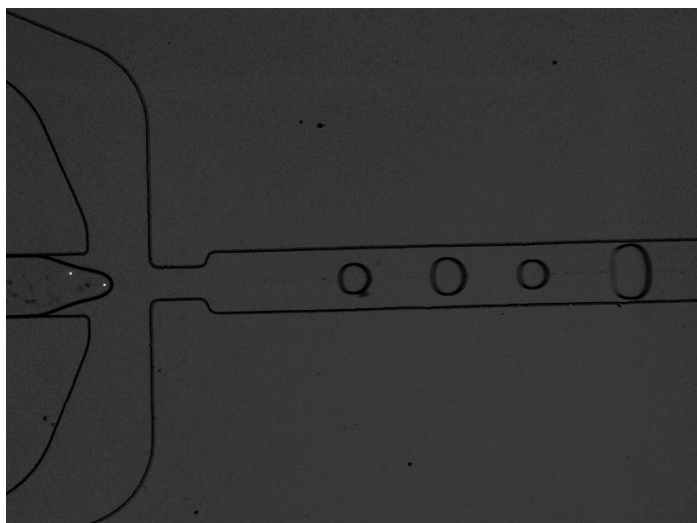

**B**

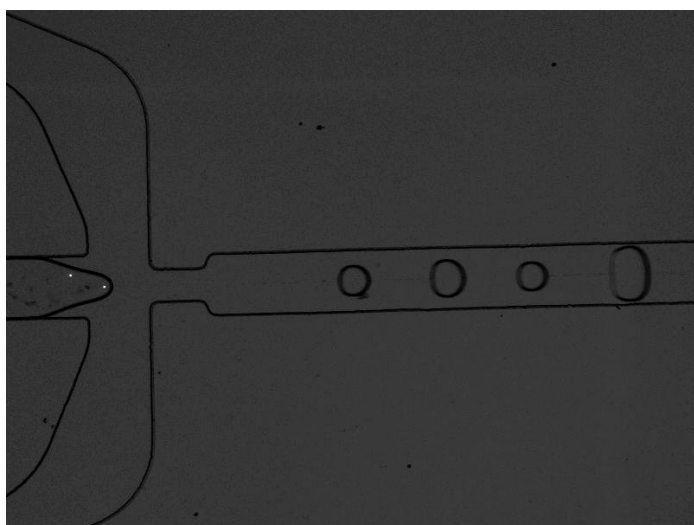

Supplement: Supplementary file 1 [file Supplementaryfile1.zip › Supplementary File 1/Supplementary Figure 2.pdf]

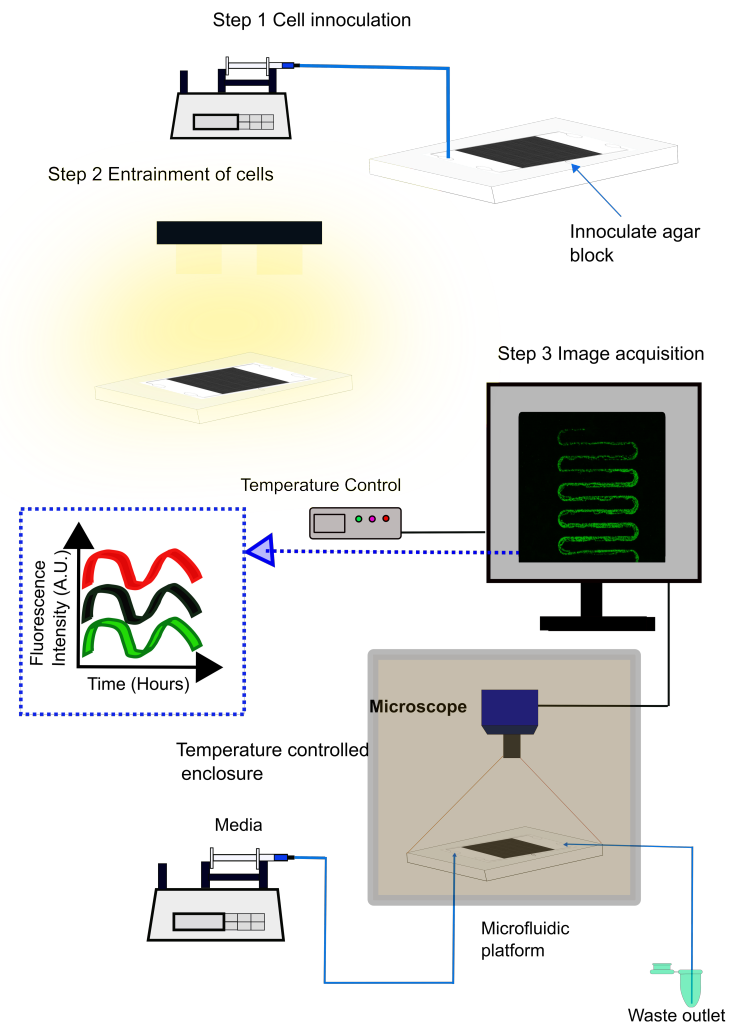

Supplement: Supplementary file 1 [file Supplementaryfile1.zip › Supplementary File 1/Supplementary Figure 4.pdf]

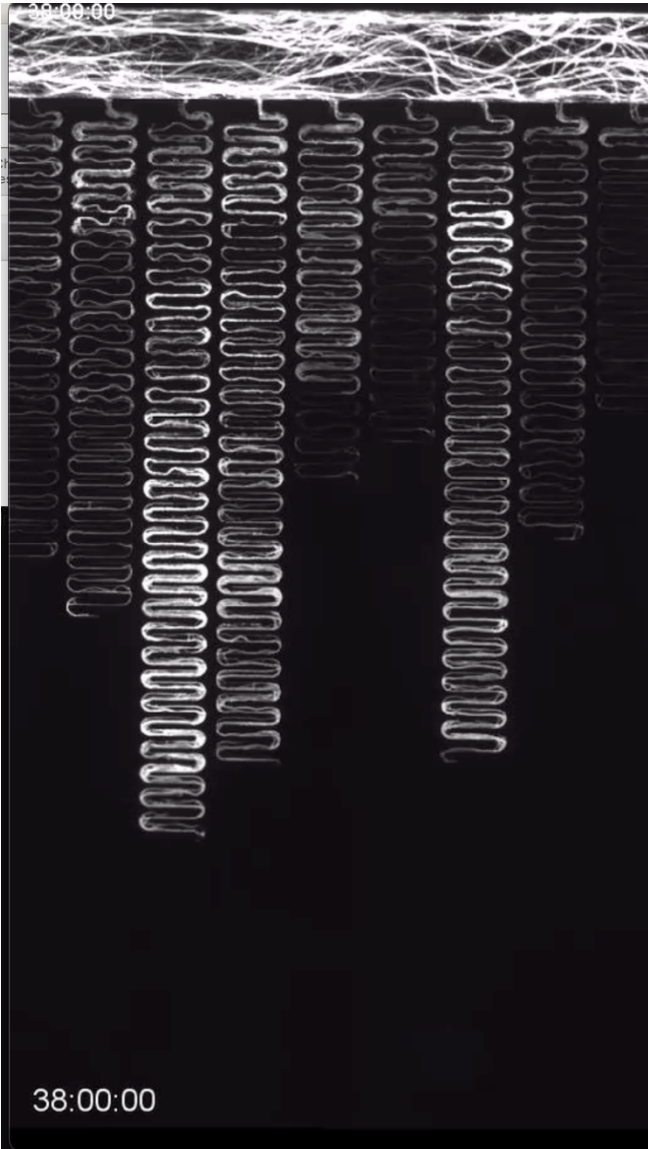

Supplement: Supplementary file 1 [file Supplementaryfile1.zip › Supplementary File 1/Supplementary Figure 5.pdf]

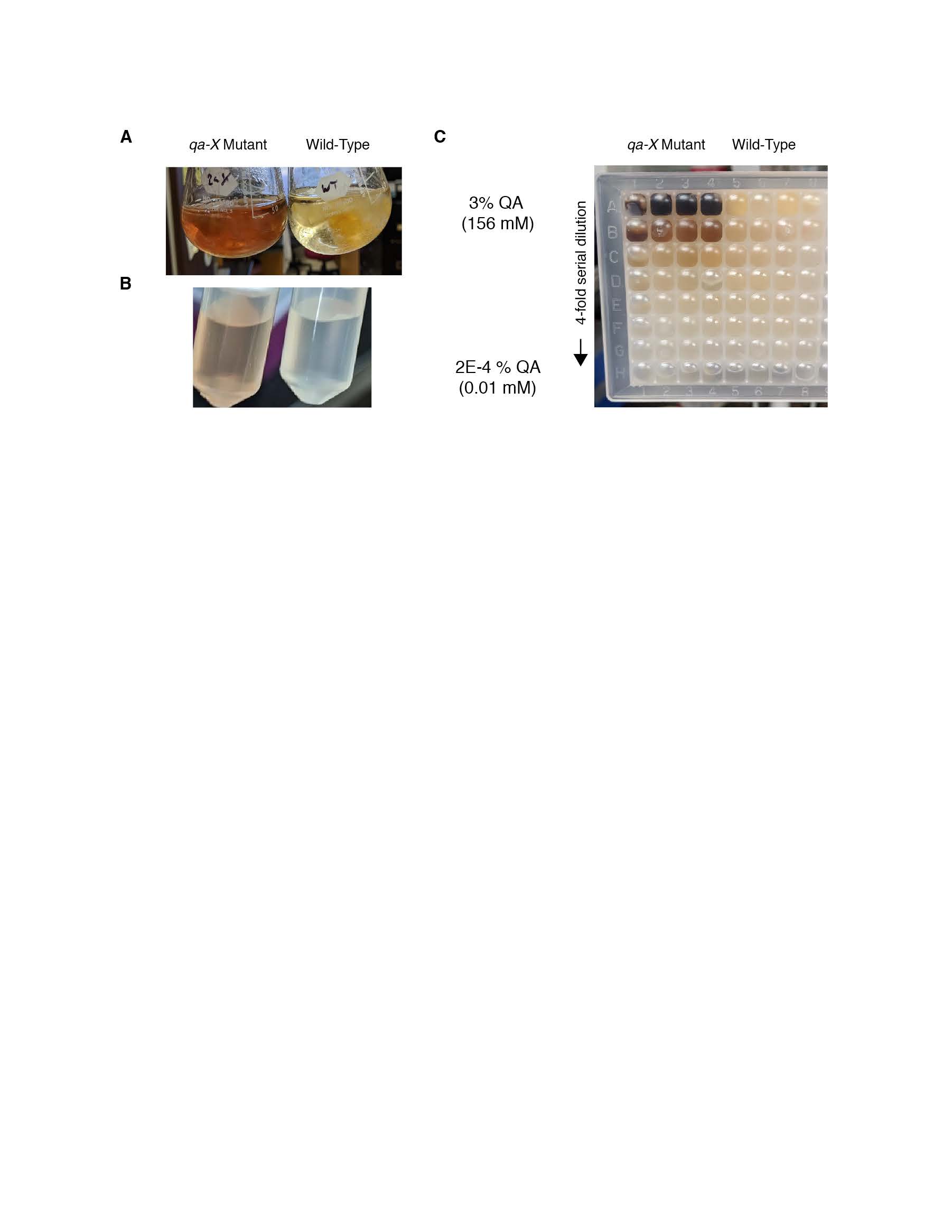

Supplement: Supplementary file 1 [file Supplementaryfile1.zip › Supplementary File 1/Supplementary Figure 6.jpg]

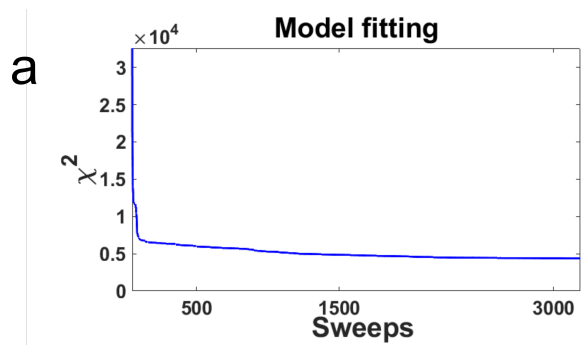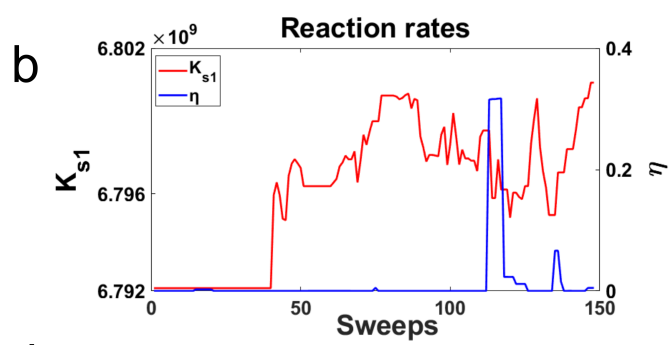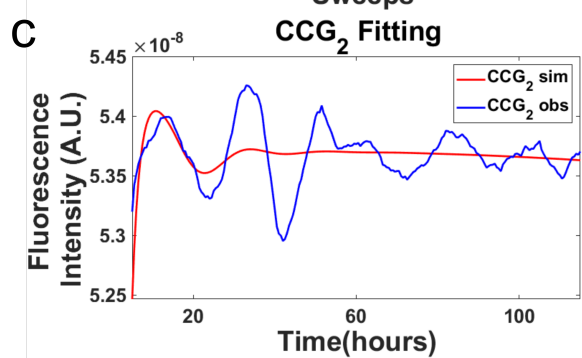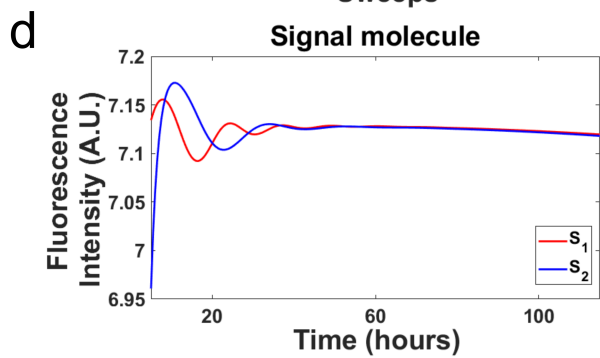

Supplement: Supplementary file 1 [file Supplementaryfile1.zip › Supplementary File 1/Supplementary Figure 8.pdf]

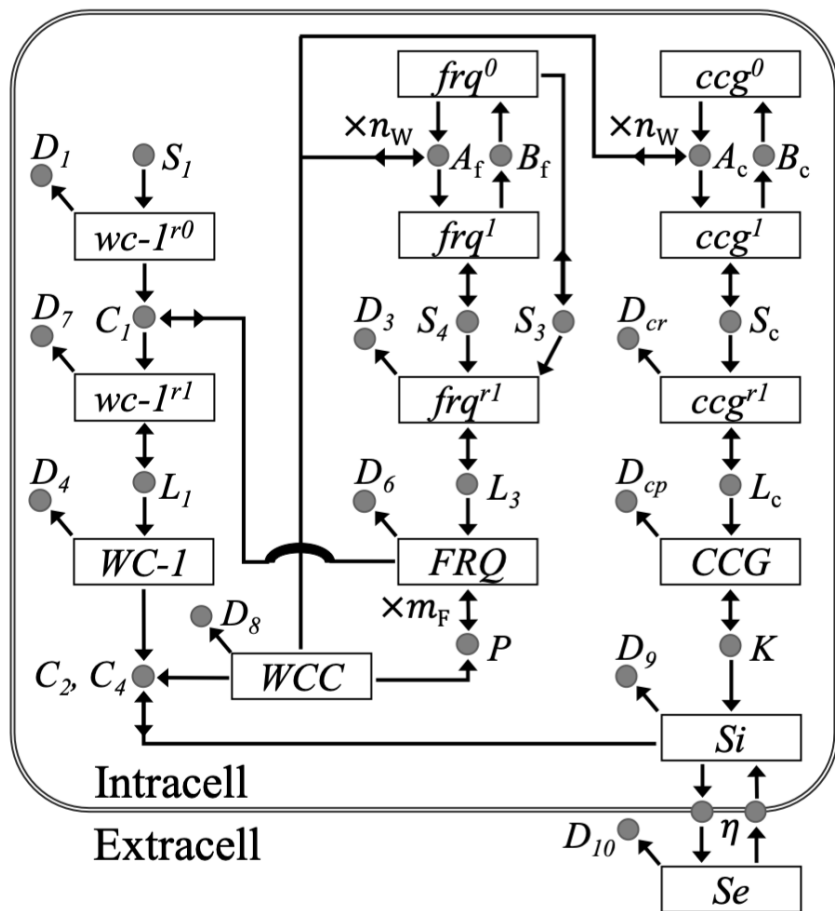

Supplement: Supplementary file 1 [file Supplementaryfile1.zip › Supplementary File 1/Supplementary Figure 9.pdf]
